# RECON syndrome is a genome instability disorder caused by mutations in the DNA helicase RECQL1

**DOI:** 10.1172/JCI147301

**Published:** 2022-03-01

**Authors:** Bassam Abu-Libdeh, Satpal S. Jhujh, Srijita Dhar, Joshua A. Sommers, Arindam Datta, Gabriel M.C. Longo, Laura J. Grange, John J. Reynolds, Sophie L. Cooke, Gavin S. McNee, Robert Hollingworth, Beth L. Woodward, Anil N. Ganesh, Stephen J. Smerdon, Claudia M. Nicolae, Karina Durlacher-Betzer, Vered Molho-Pessach, Abdulsalam Abu-Libdeh, Vardiella Meiner, George-Lucian Moldovan, Vassilis Roukos, Tamar Harel, Robert M. Brosh, Grant S. Stewart

**Affiliations:** 1 Department of Pediatrics and Genetics, Makassed Hospital and Al-Quds Medical School, East Jerusalem, Palestine.; 2 Institute for Cancer and Genomic Sciences, University of Birmingham, Birmingham, United Kingdom.; 3 Translational Gerontology Branch, National Institute on Aging, NIH, Baltimore, Maryland, USA.; 4 Institute of Molecular Biology (IMB), Mainz, Germany.; 5 Department of Biochemistry and Molecular Biology, The Pennsylvania State University College of Medicine, Hershey, Pennsylvania, USA.; 6 Department of Genetics and; 7 Paediatric Dermatology Service, Department of Dermatology, Hadassah Medical Center, Faculty of Medicine, Hebrew University of Jerusalem, Jerusalem, Israel.

**Keywords:** Cell Biology, Genetics, DNA repair, Genetic diseases, Genetic instability

## Abstract

Despite being the first homolog of the bacterial RecQ helicase to be identified in humans, the function of RECQL1 remains poorly characterized. Furthermore, unlike other members of the human RECQ family of helicases, mutations in RECQL1 have not been associated with a genetic disease. Here, we identify 2 families with a genome instability disorder that we have named RECON (RECql ONe) syndrome, caused by biallelic mutations in the *RECQL* gene. The affected individuals had short stature, progeroid facial features, a hypoplastic nose, xeroderma, and skin photosensitivity and were homozygous for the same missense mutation in RECQL1 (p.Ala459Ser), located within its zinc binding domain. Biochemical analysis of the mutant RECQL1 protein revealed that the p.A459S missense mutation compromised its ATPase, helicase, and fork restoration activity, while its capacity to promote single-strand DNA annealing was largely unaffected. At the cellular level, this mutation in RECQL1 gave rise to a defect in the ability to repair DNA damage induced by exposure to topoisomerase poisons and a failure of DNA replication to progress efficiently in the presence of abortive topoisomerase lesions. Taken together, RECQL1 is the fourth member of the RecQ family of helicases to be associated with a human genome instability disorder.

## Graphical abstract

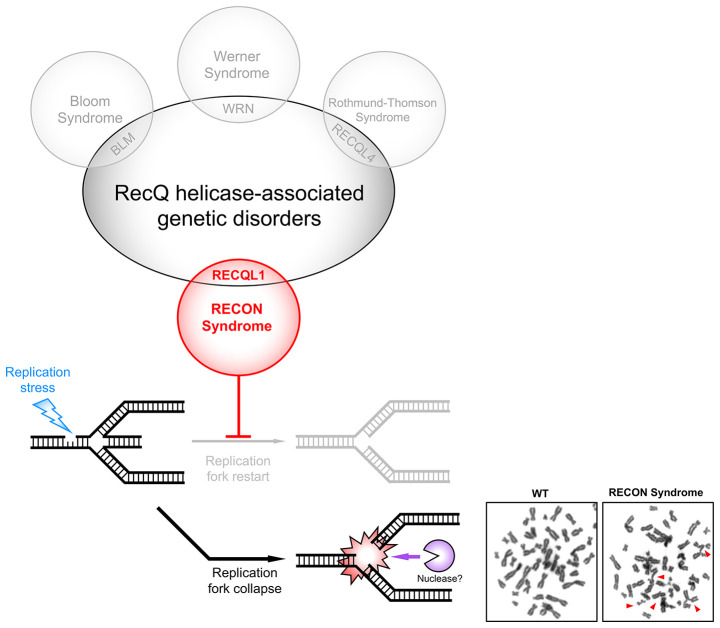

## Introduction

DNA helicases are ubiquitous enzymes found in most uni- and multicellular organisms and function to unwind DNA in an ATP-dependent and direction-specific manner. The ability to unwind DNA is an essential step in many fundamental cellular processes, such as DNA replication, recombination, repair, and transcription. The RecQ family of helicases represents one of the most highly conserved groups of 3′–5′ DNA helicases and is named after the prototype *Escherichia coli* RecQ. Although bacteria and yeast only express one RecQ-type helicase, higher eukaryotes possess multiple homologs that are highly conserved. The RecQ helicase family has 5 known homologs in humans: RECQL (also known as RECQ1 or RECQL1), WRN, BLM, RECQL4, and RECQL5. Strikingly, biallelic mutations in 3 of 5 of these RecQ homologs — *WRN*, *BLM*, and *RECQL4* — have been associated with rare genetic disorders in humans, characterized by chromosomal instability and cancer predisposition (1, 2). Notably, however, these genetic disorders are distinct, with each syndrome exhibiting a unique set of clinical and cellular features. This serves to highlight that although each of these enzymes is structurally and functionally related, they perform distinct tasks within the cell.

*WRN* is mutated in Werner syndrome (WS), which is a progeroid syndrome associated with premature aging, type 2 diabetes, osteoporosis, cataracts, graying and loss of hair, skin atrophy, and cancer predisposition. Cells from individuals with WS characteristically undergo premature replicative senescence associated with increased telomere erosion and have been reported to be hypersensitive to replication stress–inducing genotoxic agents, such as camptothecin (CPT), methylmethane sulfonate (MMS), and G-quadruplex (G4) stabilizers (3). In contrast, Bloom syndrome (BS), which is caused by mutations in *BLM*, is a developmental disorder that typically includes microcephaly, short stature, immunodeficiency, skin hyper- or hypopigmentation, photosensitivity, facial erythema, and an increased predisposition for developing cancer (4). The BLM helicase has been implicated in repairing DNA double-stranded breaks (DSBs), restarting damaged replication forks, and resolving homologous recombination (HR) intermediates, including those present in mitosis as a result of underreplicated DNA transiting through the cell cycle (5). Consequently, cells devoid of BLM are hypersensitive to replication stress–inducing genotoxins such as camptothecin (CPT), hydroxyurea (HU), and aphidicolin (APH) and exhibit a characteristic increase in spontaneous sister chromatid exchanges (SCEs). Last, mutations in *RECQL4* have been associated with 3 distinct, albeit overlapping, genetic disorders: Rothmund-Thomson syndrome (RTS), RAPADILINO syndrome, and Baller-Gerold syndrome (BGS). While patients with these disorders display some unique clinical features, e.g., poikiloderma (RTS) or craniosynostosis (BGS), the vast majority of affected individuals exhibit a constellation of developmental abnormalities, such as growth retardation, aplastic/absent thumbs, aplastic/absent patellae, aplastic/absent bones in the forearms, skin hypo-/hyperpigmentation, cleft palate, and an increased predisposition for developing cancer (6). Unlike WRN or BLM, RECQL4 is a constitutive component of the replication machinery involved in regulating replication origin firing (7). However, RECQL4 has also been suggested to play a role in repairing oxidative base damage and DSBs by HR (8, 9). Accordingly, cells lacking RECQL4 function are hypersensitive to both DSB- and replication stress–inducing agents, e.g., ionizing radiation (IR), CPT, HU and H_2_O_2_.

Although RECQL1 was the first RecQ family helicase to be identified in humans, it remains the most functionally enigmatic (10). In vitro RECQL1 can unwind a variety of DNA structures in a 3′–5′ direction, particularly those with a 3′ ssDNA tail, e.g., replication fork structures. It can also catalyze branch migration of Holliday junctions and D-loops, unwind G4 structures, and promote single-strand DNA annealing (SSA). However, unlike other human RecQ helicases, it acts poorly to unwind RNA:DNA hybrids or displace Rad51 bound to ssDNA (11, 12). Despite its demonstrated biochemical activities, mice devoid of this enzyme are phenotypically normal (13). To date, there are reports suggesting that RECQL1 is required to repair DSBs induced by IR, CPT, and etoposide (ETOP) (13, 14), although the underlying mechanism is not clear. More recently, it has been proposed that a major function of RECQL1 is to promote the restart of stalled replication forks induced by the inhibition of topoisomerase 1 and that this activity is inhibited by poly-ADP-ribose polymerase 1 (PARP1), allowing the damaged forks time to repair (14). However, whether the role of RECQL1 in promoting the restart of reversed forks caused by abortive topoisomerase activity is solely dependent on PARP1 or whether RECQL1 plays a more fundamental role in this process remains to be clarified.

While *RECQL* has not been previously associated with a defined human chromosomal instability disorder, monoallelic germline mutations have been associated with a moderately increased risk of breast cancer (15, 16). Interestingly, Sun et al. (16) identified two *BRCA1/2*-negative breast cancer families with missense variants in the zinc binding domain (ZBD) of RECQL1 (p.Arg455Cys and p.Met458Lys) that completely abolished its helicase activity. Moreover, functional analysis of conserved cysteine residues within this domain demonstrated that cysteine residues 453, 475, and 478 are critical for RECQL1 to hydrolyze ATP and unwind dsDNA but not to promote SSA (17). Combined, these observations serve to highlight the functional significance of the ZBD of RECQL1 and its importance in relation to its ability to act as a DNA helicase.

Here, we demonstrate that biallelic *RECQL* mutations are associated with a human genome instability syndrome, which we propose to name RECON (RECql ONe) syndrome. Notably, individuals from both affected, but seemingly unrelated, families carried a homozygous missense mutation (p.Ala459Ser, referred to as p.A459S) in the ZBD of RECQL1. Clinically, this *RECQL* mutation was associated with a distinct disorder characterized by striking progeroid facial features, a tiny, pinched nose, apparent skin photosensitivity, xeroderma, and slender, elongated thumbs. Functionally, the p.A459S mutation significantly compromised the ATPase/helicase activity of RECQL1 and modestly affected its ability to bind DNA. However, its capacity to promote SSA was unaffected. At the cellular level, this mutation gave rise to a modest defect in repairing abortive topoisomerase 1 (TOP1) and topoisomerase 2 (TOP2) cleavage complexes and a reduced ability to replicate in the presence of CPT- or ETOP-induced DNA damage. Furthermore, cells expressing the p.A459S mutant RECQL1 also failed to efficiently restart replication following exposure to HU and MMS, indicating that the role that RECQL1 plays in restarting damaged replication forks is not just limited to those associated with abortive TOP1/2 complexes. Taken together, these data suggest that, while it is not essential (13), RECQL1 has nonredundant functions with other RecQ helicases and that its loss compromises genome stability and normal embryonic development.

## Results

### Clinical phenotype of individuals affected by RECQL1 mutations.

**Figure 1:**
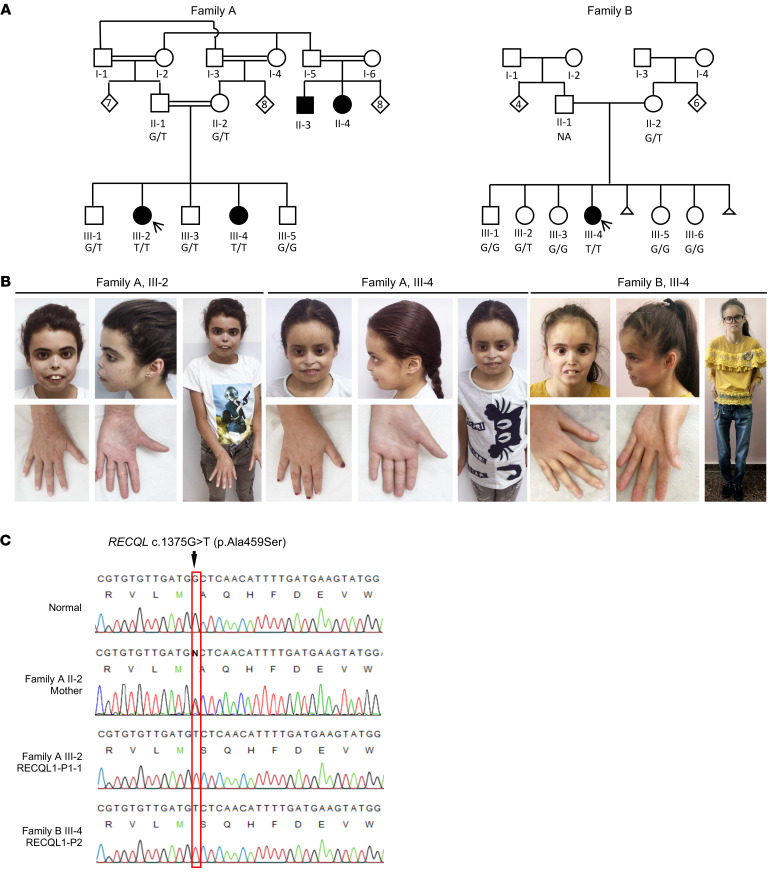
Genealogy of patients with biallelic mutations of *RECQL*. (**A**) Pedigree of the 2 families with identified mutations of *RECQL*. Patients III-2 and III-4 from family A and patient III-4 from family B are homozygous for the *RECQL* c.1375G>T (NM_032941.2) mutation. Parents and unaffected siblings from both families were either heterozygous or WT. Where samples were available, the genotypes are shown (G/G, WT; G/T, heterozygote; T/T, homozygote). Patients II-3 and II-4 from family A were reported to have a clinical phenotype similar to that of patients III-2 and III-4. The black arrow indicates probands from whom cell lines were generated: patient III-2 from family A (RECQL1-P1-1) and patient III-4 from family B (RECQL1-P2). The small triangle indicates a pregnancy not carried to term. (**B**) Photographs of the affected patients from both families showing distinctive facial characteristics and slender, elongated thumbs. (**C**) Sequencing chromatograms showing the presence of the homozygous c.1375G>T *RECQL* mutation in cDNA derived from the affected patients III-2 (family A) and III-4 (family B) and that the mother from family A (II-2) is heterozygous for the c.1375G>T *RECQL* mutation. The WT *RECQL* sequence across the position of the mutation is shown.

The proband of family A (family A, III-2) was a female evaluated at age 9 years, 8 months for short stature, dysmorphism, and dryness of the skin and eyes. She was the second of 5 children born to parents of Middle Eastern descent, who were double first cousins (Figure 1A). The pregnancy and delivery were uncomplicated. The proband was born at term with a birth weight of approximately 3000 grams. Her developmental milestones, including motor, speech, and communication skills, were age appropriate. At 18 months of age, medical evaluation was sought, since her nose remained exceptionally small compared with the rest of her face, her eyes remained open while sleeping, and she had xerophthalmia (dry eyes) and xeroderma (dry skin) with scaling. Eruption of the proband’s primary teeth was age appropriate, however, eruption of her permanent teeth was delayed. The parents reported that their child had photosensitivity. Physical examination of the girl at 9 years, 8 months revealed a height of 123.8 cm (*z* score, –1.98), a weight of 22.5 kg (*z* score, –2.05), and a head circumference of 50 cm (*z* score, –1.64). A negative *z* score represents the number of standard deviations below the population mean for each clinical measurement. The proband’s dysmorphic features included a round face, redness of eyes, a tiny, pinched nose with anteverted nares, prominent premaxilla, and a smooth philtrum (Figure 1B). Her thumbs were slender and elongated, with hyperconvex nails. She had slight hyperextension of the elbows but no joint laxity in the fingers. The proband also had livedo reticularis and keratosis pilaris (Table 1).

A younger sister of the proband (family A, III-4), examined at 4 years of age, presented with similar clinical features. The pregnancy and delivery were uncomplicated, and the girl’s developmental milestones were age appropriate. Physical examination revealed a height of 96.4 cm (*z* score, –1.02), a weight of 13.9 cm (*z* score, –1.05), and a head circumference of 47.8 cm (*z* score, –1.18). She had dysmorphism including a somewhat aged (progeroid) appearance with little subcutaneous fat, red eyes, a tiny, pinched nose with anteverted nares, prominent premaxilla, and a smooth philtrum (Figure 1B). Her thumbs were long and slender with proximal insertion, and she had mild hirsutism of the back. We noted minor joint laxity of the fingers (Table 1). The family history was also positive for 2 common adult first cousins of the parents who had similar clinical features. Unfortunately, these relatives were not available for genetic evaluation or a detailed medical workup.

The proband of family B (family B, III-4) was a female and the fourth of 6 children born to parents of Middle Eastern descent with no known consanguinity (Figure 1A). She was referred for evaluation for the first time at the age of 5 years, 10 months for dysmorphism and dryness of skin. The pregnancy and delivery were uncomplicated. She was born at term, with a birth weight of 3500 grams. Her developmental milestones, including motor, speech, and communication skills, were age appropriate. Her other medical problems at that time were thrombocytopenia of undetermined cause and recurrent chest infections. The parents reported that their child was photosensitive. Physical examination revealed a height of 110 cm (*z* score, –0.7), a weight of 13.5 kg (*z* score, –3.4), and a head circumference of 47 cm (*z* score, –2.9). Dysmorphic features included a mask senile-like face (progeroid), deep-set eyes, absent lower lid eyelashes, small ears with small, attached earlobes, a prominent nasal bridge with a tiny, pinched nose with hypoplastic alae nasi, prominent premaxilla, thin lips, and a smooth philtrum (Figure 1B). The proband had bilateral arachnodactyly, and her thumbs were slender and elongated, with hyperconvex nails. She had hirsutism of the lower limbs, and we observed thin, dry skin (xeroderma) with areas of desquamation (scaling), mainly on exposed body parts, suggestive of photosensitivity. The proband had thin extremities with hypoplastic muscles and subcutaneous fat. Follow-up measurements at age 14 years, 9 months revealed a height of 138.4 cm (*z* score, –3.59), a weight of 22.1 kg (*z* score, –8.47), and a head circumference of 50.8 cm (*z* score, –2.92) (Table 1). Neither family reported any history of breast cancer or other cancers. Genetic testing included chromosome analysis, chromosome breakage studies with diepoxybutane, and Sanger sequencing for mutations in *ERCC6* and *ERCC8*, all of which were normal.

### Whole-exome sequencing identified a homozygous missense mutation of RECQL.

To identify the genetic cause of the disorder found in family A, we performed whole-exome sequencing on DNA from the proband (III-2) of family A. After exclusion of nonauthentic variants and inconsistent mutations, data mining identified a predicted pathogenic variant in *RECQL* (chr12:21626557[hg19]: NM_032941.2; c.1375G>T; p.Ala459Ser — referred to as p.A459S) as the primary candidate (Figure 1C). This variant affects a highly conserved alanine residue (GERP score, 6.04) that lies within the ZBD of the RECQL1 protein (Supplemental Figure 1, A–C; supplemental material available online with this article; https://doi.org/10.1172/JCI147301DS1). Moreover, this variant is not found in the gnomAD or Greater Middle East (GME) variome databases, nor in our local databases of approximately 10,000 exomes. It is predicted to be pathogenic by several bioinformatics algorithms including combined annotation-dependent depletion (CADD) (score, 25.9), MutationTaster (score, 1.0), PolyPhen-2 (score, 0.846), and deleterious annotation of genetic variants using neural networks (DANN) (score, 0.9977). Notably, monoallelic alteration of the adjacent amino acid 458 (p.Met458Lys) has previously been linked with an increased predisposition for developing breast cancer. Moreover, functional studies of this mutation revealed that it significantly compromised the helicase activity of RECQL1 (14). Family B was identified because of the striking similarity of clinical features exhibited by the proband (III-4) and the 2 affected siblings from family A. Segregation analysis by Sanger sequencing of genomic DNA (gDNA) from both families confirmed segregation of this variant with the clinical phenotype (Figure 1A). All 3 affected individuals (III-2 and III-4 from family A and III-4 from family B) were homozygous for this variant, whereas the available parents and 8 healthy siblings were either heterozygous or WT [the logarithm of the odds (LOD) score (θ = 0) was *Z* = 3.311].

To determine what impact the p.A459S mutation had on RECQL1 protein stability, we performed Western blotting on whole-cell extracts generated from lymphoblastoid cell lines (LCLs) derived from RECQL1 patient III-2 from family A (RECQL1-P1-1) and RECQL1 patient III-4 from family B (RECQL1-P2). We observed no obvious differences in the expression of RECQL1 protein in LCLs from the 2 affected patients when compared with the expression of RECQL1 in LCLs from 2 normal individuals (Supplemental Figure 1C). Moreover, the p.A459S mutant RECQL1 protein localized normally to the nucleus in the presence or absence of DNA damage and was still able to associate with PARP1, a known RECQL1 binding protein (ref. 14 and Supplemental Figure 1, D–F). Therefore, to gain insight into the pathogenesis of RECON syndrome, we used biochemical and cell biological approaches to study the effects of the p.A459S mutation on the enzymatic activity of RECQL1 and its roles in regulating the cellular DNA damage response.

### RECQL1-A459S displays significantly reduced DNA unwinding activity on a preferred forked duplex DNA substrate.

**Figure 2:**
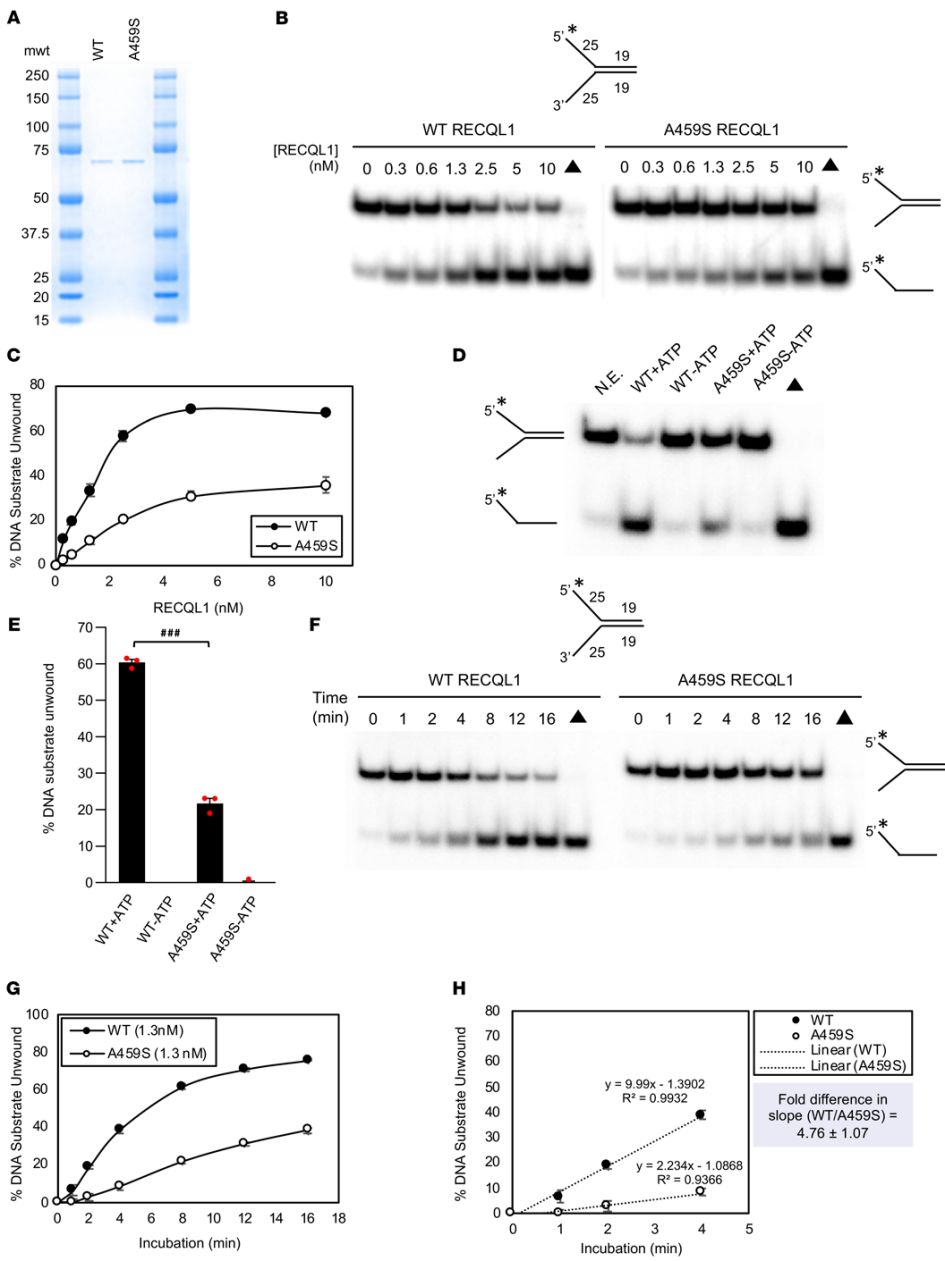
The p.A459S mutation compromises RECQL1 helicase activity. (**A**) SDS polyacrylamide gel stained with Coomassie showing recombinant human WT RECQL1 and RECQL1-A459S (500 ng each). mwt, molecular weight. (**B**) The specified RECQL1 concentrations were tested for unwinding a radiolabeled 19 bp forked duplex DNA substrate (0.5 nM) in 15 minutes. Representative gels are shown. The asterisk indicates a 5′ radiolabel on the 19 bp forked duplex DNA substrate. The black triangle indicates the heat-denatured DNA substrate control used as a marker for duplex unwinding. (**C**) Quantitation of DNA unwinding from 3 independent experiments, with the SEM shown. AUC: WT (mean ± SEM: 578.3 ± 8.01); A459S (mean ± SEM: 254.3 ± 18.63). WT versus A459S AUC: *P* < 0.0001, by unpaired *t* test. (**D**) Representative gels showing analysis of helicase activity catalyzed by RECQL1 (2.5 nM) on a 19 bp forked DNA substrate in the presence or absence of 5 mM ATP. N.E., no enzyme. (**E**) Quantitation of DNA unwinding from 3 independent experiments, with the SEM shown. ^###^*P* < 0.001, by unpaired *t* test.(**F**) Kinetics analyses of unwinding by WT RECQL1 or RECQL1-A459S. Representative gel images show analysis of reaction mixtures containing 1.3 nM WT RECQL1 or RECQL1-A459S helicase incubated with a 19 bp forked duplex DNA substrate for the indicated durations. (**G**) Quantitation of DNA unwinding from 3 independent experiments, with the SEM shown. (**H**) Quantitation of DNA unwinding showing a linear trendline and slope of unwinding from 3 independent experiments. The SEM is shown. AUC: WT (mean ± SEM: 830.3 ± 11.35); A459S (mean ± SEM: 316.1 ± 10.3). WT versus A459S AUC: *P* < 0.0001, by unpaired *t* test.

Given that it had been previously shown that mutating the residue preceding alanine-459 (methionine-458) to a lysine abolished helicase activity (16), this prompted us to biochemically characterize RECQL1-A459S and compare its properties with WT RECQL1. Initially, recombinant WT RECQL1 and mutant RECQL1 were expressed and purified from insect cells to near homogeneity (Figure 2A). Following this, using a 19 bp forked duplex as a substrate to measure its helicase activity, a simple protein titration of the WT and mutant RECQL1 proteins revealed that the p.A459S mutation substantially reduced its helicase activity. At a protein concentration of 2.5 nM, WT RECQL1 unwound nearly 60% of the DNA substrate compared with approximately 20% for mutant RECQL1 (Figure 2, B and C). To ascertain whether the DNA unwinding by RECQL1-A459S was ATP dependent, we incubated the mutant protein with the 19 bp forked duplex DNA substrate in the presence or absence of ATP. As predicted, RECQL1-A459S failed to unwind the DNA substrate when ATP was absent, consistent with the behavior of WT RECQL1 (Figure 2, D and E).

Kinetics analysis of RECQL1-dependent unwinding of the 19 bp forked DNA duplex showed a 2.6-fold reduction in the rate catalyzed by the p.A459S mutant compared with WT at a protein concentration of 0.3 nM (Supplemental Figure 2, A–C). Using a 4-fold greater concentration of RECQL1 protein (1.2 nM), the rate of helicase activity was reduced 4.8-fold for RECQL1-A459S (Figure 2, F–H), suggesting that the reduction in the unwinding rate on the short 19 bp forked duplex was in part protein concentration dependent.

RECQL1, like the other human RecQ helicases, shows significantly compromised helicase activity on longer duplex DNA substrates, suggesting that the enzyme lacks processivity (18, 19). This led us to assess helicase-catalyzed DNA unwinding by the WT and mutant RECQL1 proteins on a longer 31 bp forked duplex DNA substrate. As shown in Supplemental Figure 3, A and B, RECQL1-A459S displayed significantly reduced helicase activity on the 31 bp forked duplex compared with WT RECQL1. Approximately 3-fold less DNA substrate was unwound by RECQL1-A459S compared with WT RECQL1 at a protein concentration of 40 nM. This led us to perform a kinetics analysis of the WT and p.A459S RECQL1 proteins on the 31 bp forked duplex with 2 different concentrations of RECQL1. At a 30 nM RECQL1 concentration, we observed a 3.8-fold difference in the rate of unwinding (Supplemental Figure 3, C–E). We observed a similar reduction in the rate of unwinding by the p.A459S mutant at a RECQL1 protein concentration of 40 nM (Supplemental Figure 3, F–H).

Altogether, the findings from these helicase studies demonstrate that the p.A459S amino acid substitution in the ZBD of RECQL1 compromised its DNA unwinding activity, a result that was consistent with those of previous studies assessing the impact of other RECQL1 ZBD mutations on its helicase activity (16, 17).

### p.A459S RECQL1 poorly restores a replication fork from its regressed state.

**Figure 3:**
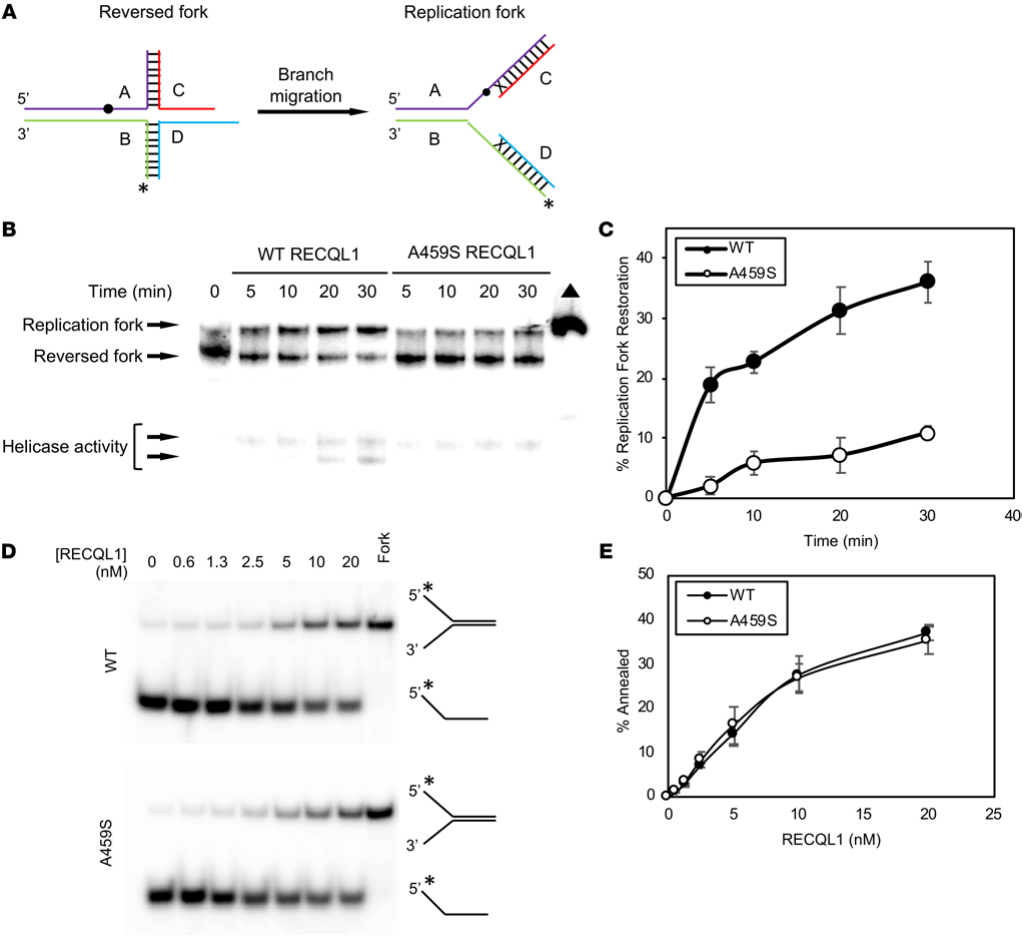
The p.A459S mutation compromises the ability of RECQL1 to remodel reversed replication forks but not its ability to catalyze SSA. (**A**) Depiction of the reversed and replication fork substrates used in this experiment. The black dot and “X” in the figure represent an isocytosine base and mismatched bases, respectively. These were added to minimize spontaneous conversion of the reversed fork into a replication fork. The asterisk indicates the 5′ end of the oligonucleotide that was radiolabeled with ^32^P. (**B**) WT or A459S RECQL1 (20 nM) was incubated with 2 nM radiolabeled reversed fork substrate at 37°C for the indicated durations. A radiolabeled replication fork (2 nM, black triangle) was included as a marker. A representative gel is shown. (**C**) Quantitation of DNA unwinding from 3 independent experiments, with the SEM shown. AUC: WT (mean ± SEM: 709.5 ± 35.23); A459S (mean ± SEM: 174.4 ± 23.91). WT versus A459S AUC: *P* < 0.0002, by unpaired *t* test. (**D** and **E**) RECQL1 strand annealing activity was assessed by the formation of a 19 bp forked duplex DNA molecule from 2 partially complementary single-stranded oligonucleotides as a function of increasing RECQL1 concentration. (**D**) Two partially complementary oligonucleotides (1 radiolabeled at the 5′ end and the other unlabeled; 0.5 nM of each was incubated with the indicated concentrations of RECQL1 for 15 minutes at 37°C in the absence of ATP). A 19 bp forked duplex was loaded as a marker. Representative gel images are shown. (**E**) Quantitation of strand annealing activity from 3 independent experiments, with the SEM shown.

Since RECQL1 has been previously shown to restore replication forks that have been regressed (14), we used a reversed fork structure (Figure 3A) as a substrate in our in vitro RECQL1 enzymatic assays to assess whether the p.A459S mutation affected the ability of RECQL1 to remodel a reversed fork. Over a 30-minute time course, the p.A459S mutant RECQL1 consistently had a reduced ability to restore the replication fork compared with WT RECQL1 (Figure 3, B and C). We observed as much as a 7-fold difference in fork restoration at the 5-minute time point and at least a 3.5-fold difference at later time points in the reaction (Figure 3, B and C). Interestingly, we also observed a small but detectable amount of helicase activity using the reversed fork structure as a substrate (Figure 3B), and this activity was also reduced by the p.A459S mutation. This indicates that the p.A459S mutation not only compromises the ability of RECQL1 to unwind forked duplex DNA structures but also to remodel reversed forks in vitro.

### RECQL1-A459S retains SSA activity comparable to that of WT RECQL1.

Purified human recombinant RECQL1 protein is also known to display potent strand annealing activity (19). To determine whether this activity is also affected by the p.A459S mutation, we examined the ability of mutant and WT RECQL1 proteins to anneal 2 partially complementary oligonucleotides to form a 19 bp forked DNA duplex. These experiments were performed in the absence of ATP to prevent helicase-catalyzed unwinding of the annealed forked duplex, which would normally serve as a canonical substrate for the RECQL1 helicase activity. As shown in Figure 3, D and E, the strand annealing activity of WT and mutant RECQL1 proteins was nearly identical over a range of protein concentrations, indicating that, despite the compromised helicase activity of RECQL1-A459S, its strand annealing activity remained intact.

### RECQL1-A459S displays modestly reduced DNA substrate binding compared with WT RECQL1.

Given that RECQL1-A459S has reduced helicase but proficient strand annealing activity compared with WT RECQL1, we wanted to examine their relative DNA binding capacities using EMSA, a sensitive technique that allows both visual evidence and quantitative assessment of nucleic acid binding. Although RECQL1 binds to various DNA substrates, it has been shown to preferentially bind a forked DNA duplex molecule over single-stranded or fully duplexed DNA molecules (11, 19). Therefore, we chose to study the DNA binding capabilities of WT and mutant RECQL1 proteins using the 19 bp forked duplex DNA substrate we had tested in the helicase assays. In this case, we omitted ATP or included ATPγS in the binding mixtures to prevent helicase activity. From these assays, we found that both mutant and WT RECQL1 proteins could bind the forked duplex DNA molecule, shifting the free radiolabeled DNA to 3 specific slower-migrating bands presumably representing the DNA substrate bound by monomeric and multimeric species (Supplemental Figure 4, A and B). However, quantitative assessment of the DNA binding isotherm data from binding incubations conducted in the absence of ATP indicated a 1.5-fold greater apparent *KD* for RECQL1-A459S compared with WT RECQL1 (Supplemental Figure 4A), suggesting a modest decrease in the affinity of RECQL1-A459S for the forked duplex DNA compared with WT RECQL1. We detected a similar increase of 1.4-fold in the *KD* value for RECQL1-A459S in the presence of ATPγS when compared with WT (Supplemental Figure 4B).

To further study the apparent difference in DNA binding between WT RECQL1 and RECQL1-A459S, we performed DNA binding assays in which a competitor oligonucleotide was used as a challenging agent after preincubation of the RECQL1 protein with the radiolabeled forked duplex DNA molecule (Supplemental Figure 5A). Both the mutant and WT RECQL1 proteins showed a similar reduction in DNA binding with an increasing concentration of unlabeled competitor (Supplemental Figure 5, B and C), suggesting that the stability of the preformed forked duplex–RECQL1 protein complex was similar for the WT and mutant forms of RECQL1.

### Effect of the RECQL1-A459S mutation on kinetic parameters for ATP hydrolysis.

To determine the ability of RECQL1-A459S to bind and hydrolyze ATP, we conducted a series of ATPase assays to measure the turnover rate constant (k_cat_), the Michaelis constant (K_m_), and the maximum velocity (V_max_) for ATP hydrolysis. In kinetics assays with an ATP concentration of 1 mM, WT RECQL1 had a nearly 3-fold higher k_cat_ for ATP hydrolysis in the presence of the M13mp18 ssDNA effector compared with RECQL1-A459S (Table 2). In ATPase assays at varying ATP concentrations, WT RECQL1 had a 3-fold greater maximum velocity (V_max_) and a 2-fold greater K_m_ compared with RECQL1-A459S (Table 2), suggesting that the diminished turnover of ATP catalyzed by the RECQL1-A459S mutant was not reflected by reduced nucleotide binding.

### RECQL1-A459S protein retains its ability to oligomerize.

To examine the effect of the RECQL1-A459S mutation on its ability to oligomerize, we performed size exclusion chromatography. As seen in previous studies (20, 21), the WT RECQL1 protein eluted from the column in 2 distinct peaks in the absence of ATP (Supplemental Figure 6). The RECQL1-A459S protein also eluted as 2 peaks at similar volumes, indicating that the mutant protein behaved similarly to the WT RECQL1 protein.

### The hypomorphic p.A459S RECQL1 mutation causes a defect in the repair of abortive TOP1/2 lesions.

**Figure 4:**
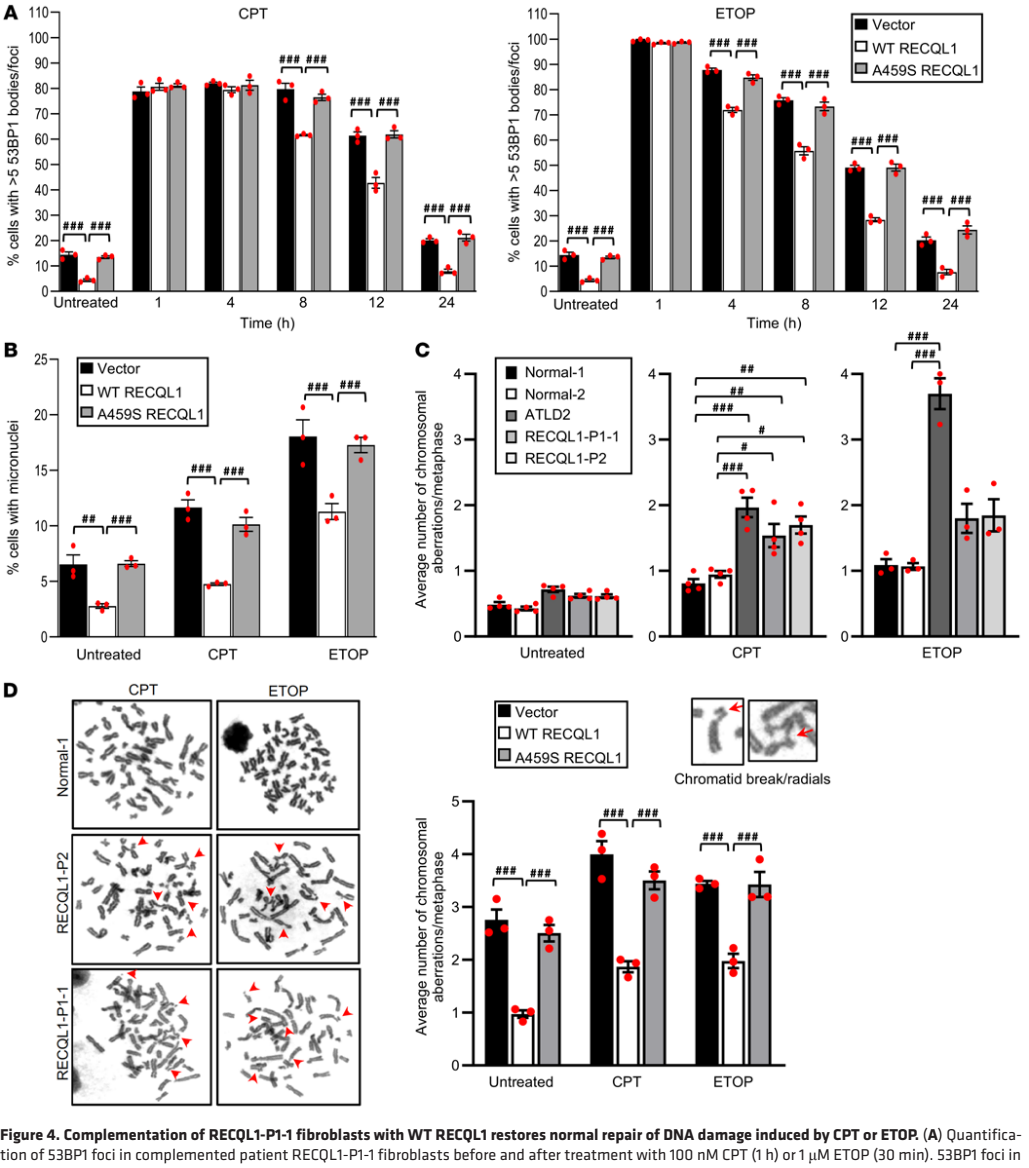
Complementation of RECQL1-P1-1 fibroblasts with WT RECQL1 restores normal repair of DNA damage induced by CPT or ETOP. (**A**) Quantification of 53BP1 foci in complemented patient RECQL1-P1-1 fibroblasts before and after treatment with 100 nM CPT (1 h) or 1 μM ETOP (30 min). 53BP1 foci in untreated cells and cells 24 hours after DNA damage induction were quantified in the G_1_ phase only (mitosin negative) to assess the amount of replication damage induced by CPT or ETOP that had transited into the following cell cycle. 53BP1 foci in cells 1 to 12 hours after DNA damage induction were quantified in the S/G_2_ phase only (mitosin positive) to monitor the kinetics of repair in damaged S/G_2_-phase cells. The mean of 3 independent experiments is shown with the SEM. A minimum of 300 cells were counted per time point. ^###^*P* < 0.001, by 2-way ANOVA with Tukey’s multiple-comparison test. (**B**) Micronuclei were quantified from cells described in **A**, before and 24 hours after exposure to CPT or ETOP. The mean of 3 independent experiments is shown with the SEM. A minimum of 500 cells were counted per time point. ^##^*P* < 0.01 and ^###^*P* < 0.001, by 2-way ANOVA with Tukey’s multiple-comparison test. (**C** and **D**) Quantification of chromosome aberrations in (**C**) 2 normal LCLs, 2 RECQL-mutant LCLs, and an ATLD LCL and (**D**) complemented patient RECQL1-P1-1 fibroblasts before and 24 hours after chronic exposure to low-dose CPT (5 nM) or ETOP (50 nM). Chromosome aberrations include chromatid/chromosome gaps and breaks, chromatid/chromosome fragments, and chromosome radials and exchanges. Representative images of each type of aberration are shown. Data show the mean ± SEM of 3 independent experiments. A minimum of 50 metaphases were counted per cell line in each experiment. ^#^*P* < 0.05, ^##^*P* < 0.01, and ^###^*P* < 0.001, by 2-way ANOVA with Tukey’s multiple-comparison test (**C** and **D**).

Since RECQL1 was previously shown to be important for the repair of DNA damage caused by poisoning TOP1/2 (14, 22), and since the p.A459S mutation significantly compromised the helicase activity of RECQL1 (this study), it was conceivable that cell lines derived from the affected patients would exhibit aberrant repair of DNA breaks induced by exposure to CPT or ETOP. To investigate this, a skin fibroblast cell line was derived from patient III-2 from family A (named RECQL1-P1-1), immortalized with hTERT, and complemented with either an empty vector or a vector expressing WT or p.A459S-mutant RECQL1. We verified the expression of the exogenous RECQL1 by Western blotting (Supplemental Figure 7A). Following this, we exposed the cell lines to low-dose CPT or ETOP for a short period of time and then incubated them in drug-free media to allow time for repair to occur. To monitor DNA DSB induction and repair, we quantified 53BP1 foci over a 24-hour time course (Figure 4A and Supplemental Figure 7B). Consistent with a role for RECQL1 in the repair of TOP1/2-associated DSBs, patients’ cells expressing either the empty vector or the p.A459S-mutant RECQL1 had higher numbers of 53BP1 foci in cells throughout a 24-hour time course following exposure to CPT or ETOP than did cells expressing WT RECQL1, despite equal levels of DNA damage being induced in all 3 cell lines and no obvious differences in the cell-cycle profile observed throughout this time course (Figure 4A and Supplemental Figure 7C). Interestingly, even in the absence of DNA-damaging agents, cells containing the empty vector or expressing the p.A459S RECQL1 mutant exhibited higher numbers of spontaneous 53BP1 foci compared with cells expressing WT RECQL1 (Figure 4A and Supplemental Figure 7B). To further validate these observations, we quantified the formation of micronuclei as a marker of genome stability in all 3 cell lines, before and after exposure to CPT or ETOP. In keeping with the 53BP1 quantification results, cells containing the empty vector or expressing p.A459S-mutant RECQL1 exhibited significantly greater numbers of spontaneous and CPT- or ETOP-induced micronuclei than did their WT RECQL1–expressing counterparts (Figure 4B). Consistent with this, RECQL1 CRISPR-KO HeLa cells complemented with empty vector or mutant RECQL1 showed a similar increase in residual 53BP1 foci and micronuclei 24 hours after exposure to CPT or ETOP when compared with KO cells complemented with WT RECQL1 (Supplemental Figure 8, A–C). However, under the growth conditions used in this study, RECQL1-KO HeLa cells did not show significant hypersensitivity to either CPT or ETOP as assessed by a colony survival assay (Supplemental Figure 8D, 8E), suggesting that more sensitive methods of measuring DNA damage repair are required to assess the impact of RECQL1 deficiency on genome stability.

To evaluate more directly what affect the p.A459S RECQL1 mutation had on DNA repair and genome stability, we quantified chromosome breakage by analysis of metaphase spreads from LCLs derived from patients RECQL1-P1-1 and RECQL1-P2. In keeping with our previous observations quantifying 53BP1 foci, the RECQL1-P1-1 and RECQL1-P2 LCLs had higher levels of spontaneous and CPT- or ETOP-induced chromosome breakage than did 2 normal LCLs. Notably, the chromosome breakage phenotype observed in the 2 RECQL1-mutant LCLs was comparable to that seen in a LCL derived from a patient with ataxia telangiectasia–like disorder (ATLD) carrying a hypomorphic homozygous truncating mutation in *MRE11A* (22), following exposure to CPT, but was significantly lower than that seen in the ATLD cell line following exposure to ETOP (Figure 4C). We observed a similar pattern when quantifying spontaneous and CPT- or ETOP-induced chromosome radial exchanges in these cell lines (Supplemental Figure 9A). Importantly, both the increased spontaneous and CPT- or ETOP-induced chromosome breakages were also observed in fibroblasts from patient RECQL1-P1 and could be complemented by the expression of exogenous WT but not mutant A459S RECQL1 (Figure 4D).

While the precise mechanism in which RECQL1 functions to mediate DSB repair is unclear, several reports have suggested that, like other RecQ helicases, RECQL1 regulates HR by affecting the interaction of the strand exchange protein Rad51 with DNA during repair (13, 23). To ascertain whether the underlying DNA repair defect in patient-derived cell lines could be caused by aberrant regulation of Rad51, we exposed the isogenic complemented fibroblasts to CPT or ETOP and then fixed and stained them with antibodies against Rad51 and mitosin (a marker of S/G_2_ cells). Quantification of Rad51 foci in S/G_2_ cells by fluorescence microscopy revealed that, although the cells containing an empty vector or expressing mutant RECQL1 had higher levels of spontaneous Rad51 than did the cells complemented with WT RECQL1 (Supplemental Figure 9B), we observed no difference between the 3 cell lines in the percentage of S/G_2_ cells with more than 10 Rad51 foci following exposure to either CPT or ETOP at both early and late time points (Supplemental Figure 9C). Consistent with the increased spontaneous Rad51 foci in the RECQL1-mutant fibroblasts, LCLs derived from both RECQL1-P1-1 and RECQL1-P2 exhibited a mild increase in spontaneous and DNA damage–induced sister chromatid exchanges (Supplemental Figure 9D). Similar observations were reported for *Recql*-KO mouse embryo fibroblasts exposed to ionizing radiation (13). Importantly, this phenotype was recapitulated in the RECQL1 CRISPR-KO HeLa cells and could be complemented by reexpression of WT but not p.A459S-mutant RECQL1 (Supplemental Figure 9E). Taken together, these results suggest that the defective repair of CPT- or ETOP-induced DNA damage may arise because of defective DNA damage response (DDR) signaling and/or an inability of the replisome to progress past these lesions, resulting in the transiting of underreplicated DNA through the cell cycle to the following G_1_ phase. To investigate the first possibility, LCLs from 2 normal individuals and the 2 affected RECQL1-mutant patients (RECQL-P1-1 and RECQL1-P2) were transiently exposed to CPT or ETOP, and, following the induction of DNA damage, early and late time points were set to examine the activation of the DDR by Western blotting using phosphorylated ATM (p-ATM), p-SMC1, p-Nbs1, p-Chk1, p-RPA2, and p-H2AX as markers. Despite some mild variability in the level of the DDR induced by CPT or ETOP, both RECQL1-mutant cell lines showed robust activation of the ATM-dependent DDR (Supplemental Figure 10), suggesting that defective DDR signaling is not the underlying cause of compromised repair of CPT- or ETOP-induced DNA lesions in RECQL1-mutant cells.

### An inability to repair TOP2-associated DSBs in cells derived from patients with RECON syndrome does not compromise the induction or repair of DSBs within the MLL locus.

It is known that TDP2, MRE11, and components of the NHEJ DSB repair pathway are critical for the repair of TOP2-induced DSBs, especially within the *MLL* locus, which frequently undergoes chromosomal translocation if misrepaired (24, 25). Given the inability of cells from patients with RECON syndrome to efficiently repair ETOP-induced DSBs, we sought to investigate whether the hypomorphic p.A459S RECQL1 mutation present in these cells was associated with aberrant repair of DSBs within the *MLL* locus. Using C-Fusion 3D, a high-throughput imaging methodology to probe chromosomal breakage and rare translocations in single cells (25), we exposed 2 normal LCLs, LCLs from a patient with a TDP2 mutation, and the LCLs from 2 patients with RECON syndrome to ETOP and monitored *MLL* locus breakage and translocation to the *ENL* locus. Notably, while the TDP2 mutant LCL displayed a high frequency of breakage of the *MLL* gene locus and translocation to the *ENL* locus following exposure to ETOP, we did not observe this in either of the RECQL1-mutant LCLs (Supplemental Figure 11, A and B). These results suggest that RECQL1 does not play a major role in the repair of TOP2-dependent DSBs located within the *MLL* locus.

### Cells from patients with RECON syndrome exhibit a reduced ability to replicate in the presence of abortive topoisomerase lesions.

**Figure 5:**
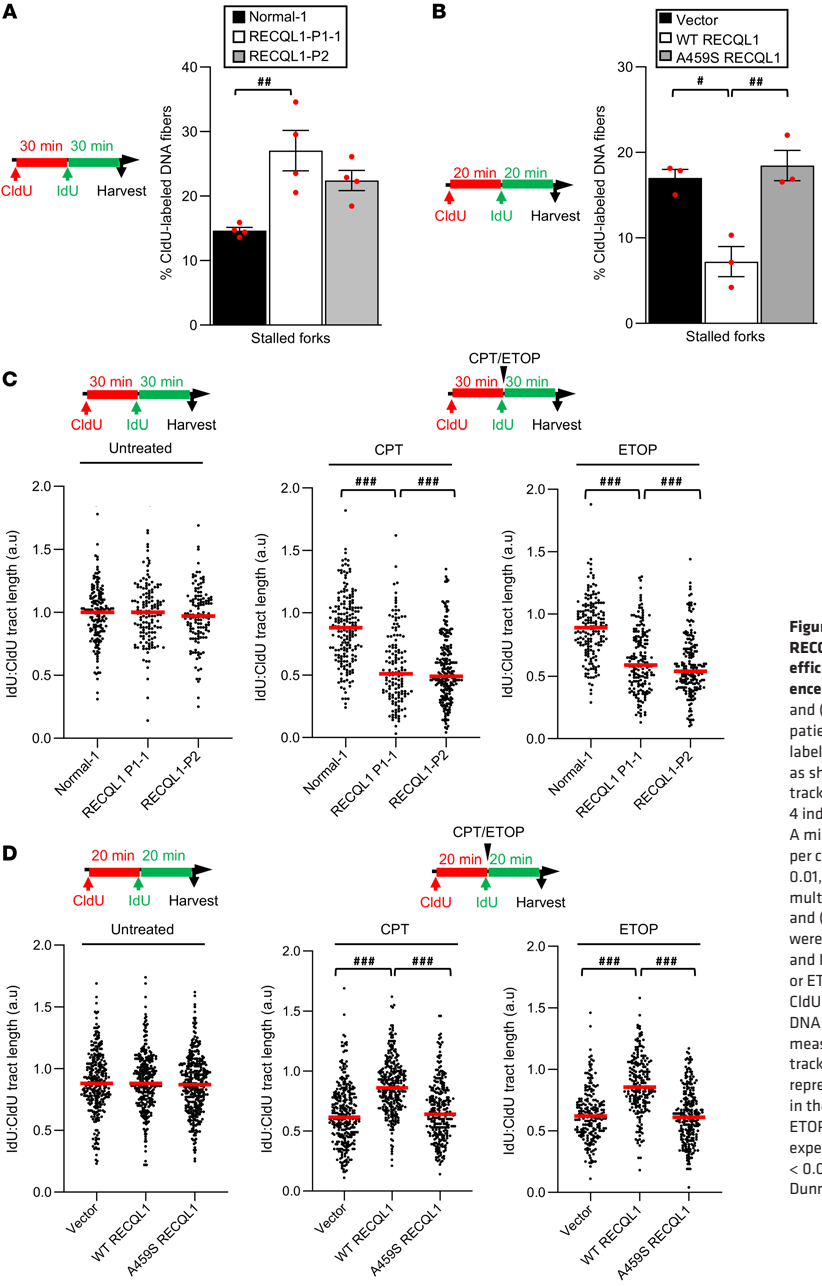
Cells from a patient with RECON syndrome display a reduced efficiency of replication in the presence of TOP1/2 inhibitors. (**A**) LCLs and (**B**) complemented RECQL1-P1-1 patient fibroblasts were sequentially labeled with CldU (red) and IdU (green) as shown. Stalled forks (red-only tracks) were quantified. The mean of 4 independent experiments is shown. A minimum of 200 forks were counted per condition. ^#^*P* < 0.05 and ^##^*P* < 0.01, by 1-way ANOVA with Tukey’s multiple-comparison test. (**C**) LCLs and (**D**) complemented fibroblasts were sequentially labeled with CldU and IdU (with or without 50 nM CPT or ETOP) as shown. The length of the CldU and IdU tracks of dual-labeled DNA fibers (>150 per condition) was measured, and the ratio of IdU/CldU track length was calculated, which represents the efficiency of replication in the presence or absence of CPT or ETOP. The median of 3 independent experiments is shown (red line). ^###^*P* < 0.001, by Kruskal-Wallis test with Dunn’s multiple comparison.

It was reported that RECQL1 is required to restart stalled replication forks following the pharmacological inhibition of TOP1 and that cells lacking RECQL1 activity accumulate reversed forks in the presence of CPT (14). To investigate the impact of the RECQL1 p.A459S mutation on replication, we carried out DNA fiber analysis using LCLs from the affected RECQL1 patients in the presence or absence of CPT or ETOP. Interestingly, even in the absence of exposure to genotoxins, both RECQL1 LCLs had an increase in spontaneously stalled forks indicative of higher levels of endogenous replication stress (Figure 5A). Although our analysis cannot distinguish between stalled and reversed forks, as they would both give rise to CldU-only fibers, it is plausible that our observations are consistent with a previous study demonstrating that depletion of RECQL1 results in an increase in reversed forks, even in the absence of exogenously induced DNA damage (26, 27). Following exposure to either CPT or ETOP, our DNA fiber analysis also revealed that both patient RECQL1-mutant cell lines were unable to efficiently replicate in the presence of abortive TOP1 or TOP2-induced DNA lesions, despite the lack of gross alterations in replication fork speed or new origin firing prior to treatment (Figure 5C and Supplemental Figure 12, A and B). Notably, both the spontaneous and CPT- or ETOP-induced replication phenotypes were also observed in the patient-derived fibroblasts and could be complemented by the expression of WT, but not mutant p.A459S, RECQL1 (Figure 5, B and D, and Supplemental Figure 12, C and D). Taken together, these data suggest that the clinically relevant p.A459S mutation in the RECQL1 ZBD, which perturbs its catalytic activity, compromises the ability of RECQL1 to restart reversed replication forks.

### Cells from individuals with RECON syndrome exhibit a reduced ability to efficiently restart HU- and MMS-damaged replication forks.

**Figure 6:**
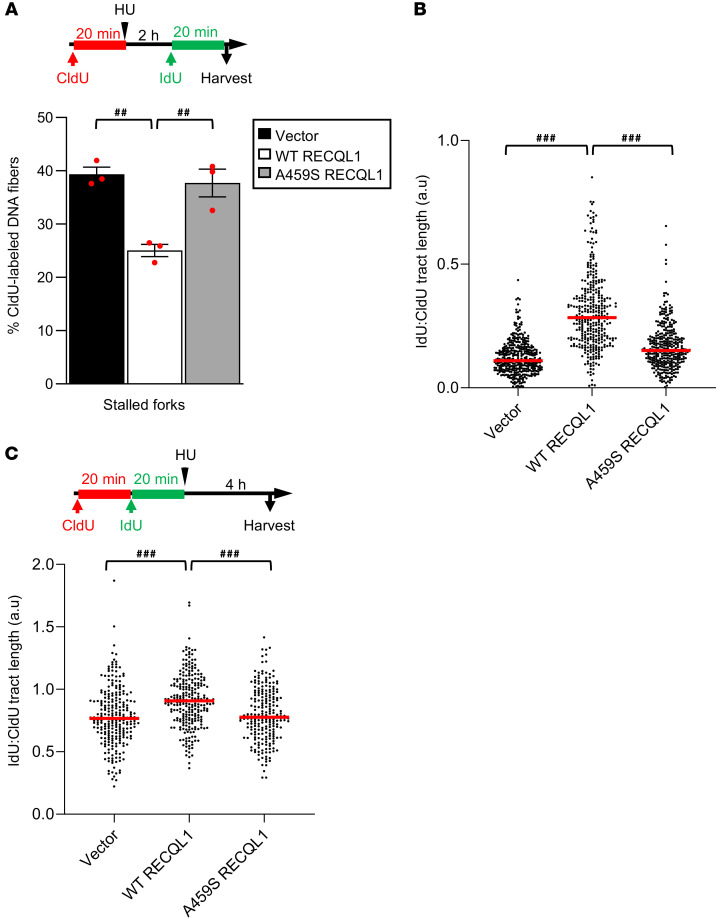
Cells from a patient with RECON syndrome exhibit reduced replication fork restart and increased replication fork degradation following exposure to HU. (**A**). Complemented RECQL1-P1-1 patient fibroblasts were labeled with CldU and IdU following exposure to 2 mM HU as shown. Stalled forks were quantified. The mean of 3 independent experiments is shown. A minimum of 200 forks were counted per condition. ^##^*P* < 0.001, by 1-way ANOVA with Tukey’s multiple-comparison test. (**B**) Cells from **A** were labeled with CldU and IdU as shown in **A**. The IdU/CldU ratio was calculated for a minimum of 100 forks per condition from 3 independent repeats. The IdU/CldU ratio represents the efficiency of replication in the absence HU and the efficiency of replication fork restart following removal of HU. (**C**) Cells from **A** were labeled with CldU and IdU and then exposed to 4 mM HU as shown. The IdU/CldU ratio was calculated for a minimum of 100 forks per condition from 3 independent experiments. An IdU/CldU ratio of less than 1 indicates replication fork degradation. ^###^*P* < 0.0001, by Kruskal-Wallis test with Dunn’s multiple comparison (**B** and **C**).

It was previously determined that RECQL1, like other helicases such as WRN (26), DNA2 (26), and BLM (28), plays a role in promoting the restart of HU-stalled forks (29). To determine whether the cell lines derived from patients with RECON syndrome exhibit a global inability to restart replication forks that is not just restricted to forks stalled by abortive topoisomerase cleavage complexes, we performed DNA fiber analysis with RECQL-P1-1–complemented fibroblasts following a transient exposure to high-dose HU. Interestingly, this analysis demonstrated that the p.A459S mutation significantly compromised the ability of replication forks transiently stalled by HU to restart efficiently (Figure 6A). Importantly, this phenotype was also recapitulated in the RECQL1 CRISPR-KO HeLa cells complemented with either an empty vector or the p.A459S RECQL1 mutant (Supplemental Figure 12E). Furthermore, we found that the efficiency of replication progression for those forks that did manage to restart following release from HU was also severely affected in the RECQL-P1-1 fibroblasts complemented with an empty vector or the p.A459S mutant compared with those complemented with WT RECQL1 (Figure 6B). These data suggest that RECQL1 may be important for the restart of all types of stalled replication forks, irrespective of the causative DNA lesion. In keeping with this, the p.A459S mutant also compromised the ability of cells to restart replication in the presence of DNA alkylation damage induced by MMS exposure, accompanied by increased chromosome breakage and cell death (Supplemental Figure 13).

Last, it has been suggested that RECQL1, in a manner similar to that of WRN and DNA2 (26), also functions to protect stalled forks from uncontrolled nucleolytic degradation. Therefore, to assess whether the inability of the p.A459S-mutant RECQL1 protein to efficiently restart HU-stalled forks is due to excessive fork degradation, we sequentially labeled the 3 isogenic complemented fibroblast cell lines with CldU and IdU and then exposed them to 4 mM HU for 5 hours. We then quantified replication fork stability by calculating the ratio of CldU- to IdU-labeled fibers. As shown in Figure 6C, RECQL-P1-1 fibroblasts complemented with empty vector and the p.A459S exhibited a mild increase in replication fork degradation after prolonged exposure to HU that could be complemented following the expression of WT RECQL1. This suggests that the replication fork restart defect present in the cells derived from patients with RECON syndrome was not only due to an inability of the mutant RECQL1 to remodel reversed forks but also resulted from the degradation of stalled forks.

## Discussion

Despite the cloning of *RECQL* over 25 years ago (10), the role that it plays within the cell to maintain genome stability remains relatively unclear. In contrast, functional insight into how other RecQ helicase family members, such as BLM, WRN, and RECQL4, protect the genome from deleterious DNA damage has been greatly facilitated by the identification of human disorders caused by mutations in these genes. However, even with the rapid advances in whole-exome/genome sequencing technology, mutations of the 2 remaining RecQ helicase genes (*RECQL* and *RECQL5*) have yet to be linked to an inherited chromosomal instability syndrome. Here, we describe the identification of a human chromosomal instability disorder, which we named RECON syndrome (RECql ONe), caused by biallelic missense mutations of *RECQL*. Notably, although it has been suggested that the function of RECQL1 may be redundant with other RecQ helicases such as BLM (30), the individuals affected by the RECQL1 p.A459S mutation exhibit a clinical phenotype distinct from that of BS, WS, and the 3 RECQL4-associated disorders, suggesting that RECQL1 is not redundant with WRN, BLM, or RECQL4 and has unique functions during human development.

Although individuals with BS or one of the RECQL4-associated disorders are clinically distinguishable, they do exhibit an overlap of symptoms, such as pre- and postnatal growth retardation, facial erythema that worsens upon sun exposure, poikiloderma, skin hypo- and hyperpigmentation, alopecia, and an increased predisposition for cancer, which is probably reflective of common cellular processes that the encoded proteins have been reported to take part in, e.g., DSB end-resection and DNA replication (7, 8, 28, 31). In comparison with the clinical features of patients with BS, RTS, RAPADILINO, or BGS, those with RECON syndrome only share postnatal growth retardation, even though RECQL1 is also implicated in regulating DSB repair and processing replication intermediates (14, 26, 29, 32).

In contrast, individuals with BS can be differentiated by the presence of microcephaly, immunodeficiency, and type 2 diabetes, whereas patients with a RECQL4-associated disorder can be identified by absent or hypoplastic thumbs, radial ray defects, absent or hypoplastic patellae, limb malformations, cataracts, reduced bone density, anemia, neutropenia, and craniosynostosis.

It appears that the defining clinical features of RECON syndrome include skin photosensitivity, xeroderma, and a progeroid-like facial appearance with a tiny, pinched nose and prominent premaxilla. Interestingly, the progeroid-like facial appearance, growth retardation, and muscle wasting are somewhat reminiscent of WS, and although it is likely that the affected patients are currently too young to ascertain whether they have any other clinical signs of premature aging, it is tempting to speculate that this represents another progeroid syndrome (33, 34).

Although it is apparent that the clinical phenotype exhibited by the affected individuals carrying *RECQL* mutations is relatively mild when compared with other RecQ-associated disorders, it should be noted that these individuals are not null for RECQL1 and that the severity of the resulting clinical symptoms is likely to be tempered by the presence of some residual protein function. In addition, it is possible that some of the features of the disease are specific to this particular *RECQL* mutation and/or were modified by the similar genetic background of the 2 affected families. As such, the range and severity of the clinical symptoms linked with RECQL1 dysfunction will only be properly determined following the identification of additional patients. In relation to this, it has been reported by 2 laboratories that heterozygous mutations of *RECQL* are associated with an increased risk of breast cancer (15, 16), although this association has been disputed by several other groups (35, 36). Interestingly, one of the identified breast cancer–associated variants in *RECQL* altered the amino acid residue proximal to the RECON syndrome *RECQL* mutation. Despite this, none of the heterozygous carriers of the p.A459S mutation in either family A or B has reported developing breast cancer. Thus, it is plausible that this *RECQL* variant might not be associated with an increased cancer predisposition, albeit the carrier frequency for the p.A459S mutation is extremely low. However, since we have not directly compared the pathogenic impact of the syndrome and cancer-associated mutations of *RECQL* on its enzymatic activities and roles in regulating DNA replication, we acknowledge that it is difficult to make any conclusions regarding whether the p.A459S mutation may predispose individuals to breast cancer. As such, further studies to address the genotype-phenotype relationship linking the molecular, cellular, and clinical characteristics of individual RECQL1 mutations are warranted.

It has been suggested that the main function of RECQL1 is to promote the restart of reversed replication forks induced by abortive TOP1-cleavage complexes, which in turn is inhibited via an interaction of RECQL1 with PARP1 (14, 26). Interestingly, we detected a replication defect in cells from patients with RECON syndrome by DNA fiber analysis following the pharmacological inhibition of either TOP1 or TOP2, without a requirement to suppress PARP1 activity. This finding suggests that RECQL1 plays a more prominent role in regulating the restart of replication forks in the presence of abortive topoisomerase complexes than was perhaps thought and that this function is only partly dependent on PARP1.

**Figure 7:**
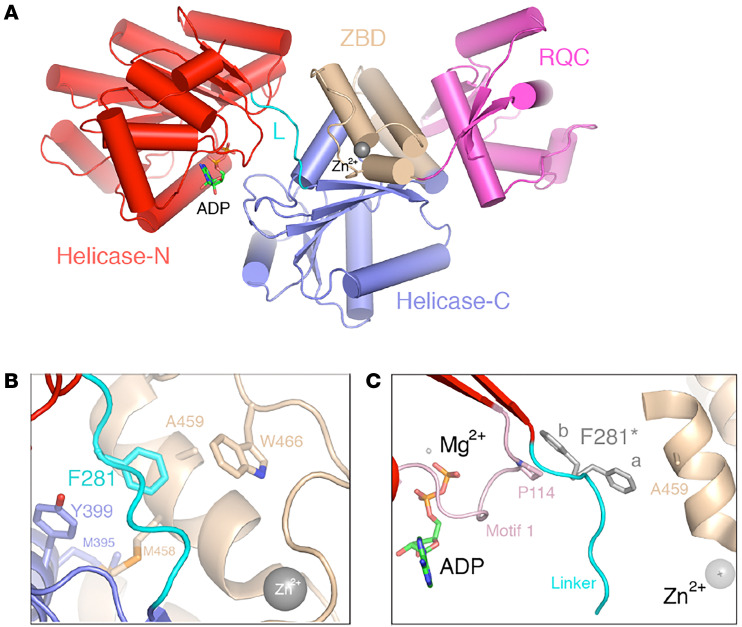
Structural modeling of the RECQL1 p.A459S mutation. (**A**) Overall structure of the RECQL1 monomer shown in a ribbons illustration, with the individual domains colored according to Supplemental Figure 1A. The gray sphere represents the Zn^2+^ ion within the ZBD, and the ADP is shown as sticks. (**B**) A459 forms part of an extended hydrophobic cluster involving W466 and M458 from the ZBD, M395 and Y359 from the helicase-C domain, and F281 from the helicase linker (cyan). (**C**) Two conformations of F281 within the helicase linker observed in the ADP-bound structure suggest a molecular basis for the dynamic coupling of structural perturbations in the ZBD to ATPase/helicase activity. The P-loop is highlighted in light pink. Conformation “b” of F281 stacks against the P144 pyrrolidine ring, while conformation “a” packs against A459. The figure was produced using PyMOL (https://pymol.org/2/) and coordinates from PDB accessions 2V1X and 2WWY (37, 47).

The ability of RECQL1 to restart forks through branch migration of a reversed fork was shown to require its ATPase activity (14). While the p.A459S mutation lies outside the RECQL1 helicase domain, it has been previously demonstrated that mutating conserved cysteine residues within the ZBD compromises both its ATPase and DNA unwinding activities (11, 12). In keeping with this, we have shown that the p.A459S mutation also substantially reduced the ATPase, DNA unwinding, and branch-migrating activities of RECQL1. Examination of the available crystal structures of human RECQL1 showed that the ZBD is spatially juxtaposed with the helicase ATP-binding cleft, which is located between the 2 N-terminal domains (Figure 7A). Interestingly, A459 contacts F281 located within the linker region between the 2 helicase domains that appears to adopt 2 conformations in the GDP-bound structure (37), one of which packs directly against P114 within the P-loop/Walker A/Motif1 region. Since A459 itself forms part of a hydrophobic cluster within the ZBD core that is likely perturbed by mutation to a more polar and bulkier serine residue, the presence of the serine residue may affect the ability of F281 to adopt these different conformations, thus potentially offering a molecular explanation for the effects of the p.A459S mutation on both ATPase and helicase activity (Figure 7, B and C). In a manner similar to the RECQL1 p.A459S mutation, disease-causing missense mutations in the ZBD of BLM also compromise its ATP hydrolysis and DNA-unwinding activities (38, 39). However, unlike RECQL1, BLM also contains a helicase and RNase C-terminal (HRDC) domain, which is required for Holliday junction dissolution (40). Therefore, even though pathogenic missense mutations have been identified in the conserved ZBD of BLM and RECQL1, the functional impact and clinical consequences of these mutations are likely to be distinct, given the unique structural architecture of the respective RecQ helicases.

Taken together, it is likely that the replication defects we observed in cells from patients with RECON syndrome are attributed to an inability of RECQL1 to restart reversed forks due to compromised ATP-dependent helicase/branch migration activity. Fork reversal is thought to be a ubiquitous response to most types of replication stress and is essential for both the repair and restart of damaged replication forks. Therefore, if RECON syndrome represents a disease of failed replication restart, it is not clear why the clinical symptoms exhibited by the affected patients do not have more of a developmental component similar, for example, to that of patients with Schimke immuno-osseous dysplasia (SIOD), which is caused by mutations in the fork remodeling factor SMARCAL1 (41, 42). This suggests that perhaps the fork restart function of RECQL1 is essential in some cell types but not in others or is restricted to a subset of forks.

Interestingly, in addition to RECQL1, other disease-associated DNA helicases involved in replication fork processing and homologous recombination, such as DNA2, WRN, BLM, FANCJ, and FANCM (43), have also been implicated in suppressing the nucleolytic degradation of stalled replication forks and promoting their restart once repaired. Notably, of these, the cellular replication response to CPT and HU damage in cells lacking WRN is most similar to that observed in RECON syndrome cells, i.e., both RECON syndrome and WS cells display increased fork degradation and a reduced efficiency with which replication can proceed in the presence of abortive TOP1 complexes or following HU exposure (26, 44). The similar cellular phenotypes of WS and RECON cells may explain the progeroid-like features of both disorders. However, the increased severity of clinical symptoms exhibited by individuals with WS when compared with patients with RECON syndrome may arise as a consequence of loss of specific enzymatic activities and/or functions of the WRN protein, e.g., the exonuclease activity, that are not conserved in RECQL1. In this regard, it remains to be determined whether RECQL1 also plays a role in promoting telomeric replication by dissembling G-quadruplex structures or repairing oxidative damage in a manner similar to WRN.

In summary, we demonstrate that mutations in *RECQL* are the underlying cause of an inherited chromosomal instability syndrome that shares some clinical and cellular phenotypic similarities with WS. Thus, to our knowledge, RECQL1 represents the fourth RecQ helicase to be implicated in genetically inherited disease characterized by genome stability defects.

## Methods

Further information can be found in Supplemental Methods.

### Exome analysis.

We performed exome analysis using DNA extracted from whole blood of the proband in family A (Figure 1A). Exonic sequences from DNA were enriched with the Agilent V5 Kit (Agilent Technologies). Sequences were generated on a HiSeq2500 (Illumina) as 125 bp paired-end runs. Read alignment and variant calling were performed with DNAnexus using default parameters with the human genome assembly hg19 (GRCh37) as a reference. Exome analysis of the proband yielded 60.95 million reads, with a mean coverage of 88.83×.

### Segregation analysis.

An amplicon containing the *RECQL* variant was amplified by conventional PCR of genomic DNA derived from the probands and all available parents and siblings. PCR amplicons were analyzed by Sanger sequencing. The genomic DNA primer sequences used for *RECQL* PCR amplification were as follows: *RECQL* forward, 5′-TTAGCCTATAAAGGTTTACAAAACA-3′ and *RECQL* reverse, 5′-GGTAATATTATGGCAGTTATAGGAAGC-3′. The primers used to sequence *RECQL* cDNA were as follows: *RECQL* forward, 5′-TGCAGGTCGAGATGACATGA-3′ and *RECQL* reverse, 5′-AGCATGTTTGCAGCCTTCTTC-3′.

### Data availability.

The *RECQL* gene variant data are available in the NCBI’s ClinVar archive (accession number SCV001364447.1).

### Cell culture and generation of cell lines.

Dermal primary fibroblasts were grown from skin-punch biopsies and maintained in DMEM (Thermo Fisher Scientific) supplemented with 20% FCS, 5% l-glutamine, and 5% penicillin-streptomycin antibiotics (Merck). Primary fibroblasts were immortalized with a lentivirus expressing human telomerase reverse transcriptase (hTERT) that was generated by transfecting 293FT cells (Thermo Fisher Scientific) with the plasmids pLV-hTERT-IRES-hygro (Addgene no. 85140), psPax2 (Addgene no. 12260), and pMD2.G (Addgene no. 12259). Selection was performed using hygromycin (Thermo Fisher Scientific) at 70 μg/mL. All LCLs were routinely grown in RPMI-1640 (Thermo Fisher Scientific) supplemented with 10% FCS, 5% l-glutamine, and 5% penicillin-streptomycin. *RECQL*-KO HeLa cells were routinely grown in DMEM supplemented with 10% FCS, 5% l-glutamine, and 5% penicillin-streptomycin. Fibroblast and HeLa cell complementation was carried out using the pLVX-IRES-Neo lentiviral vector (Takara Bio) encoding 2xHA-tagged *RECQL*. All cell lines were routinely tested for mycoplasma.

### Genotoxic agents.

Camptothecin (Merck), etoposide (Merck), hydroxyurea (MilliporeSigma), and methylmethane sulfonate (MilliporeSigma) were used as indicated.

### Immunoblot analysis, immunoprecipitation, and antibodies.

Whole-cell extracts were prepared and subjected to immunoblotting as previously described (23). Immunoblotting was performed using antibodies against pS1981 ATM (AF1655) from R&D Systems; ATM (A300-299A), pS824-KAP1 (A300-767A), KAP1 (A300-274A), pS966-SMC1 (A300-050A), SMC1 (A300-055A), pS4/S8-RPA2 (A300-245A), and RECQL1 (A300-450A) from Bethyl Laboratories; pS345-CHK1 (2341) from Cell Signaling Technology; CHK1 (sc-8408) and PARP1 (sc-8007) from Santa Cruz Biotechnology; H2A (07-146), γ-H2AX (05-636), RPA2 (NA18), and HA (H9658) from Merck; pS343-NBS1 (47272) from Abcam; and Nbs1 (GTX70224) from GeneTex. Co-immunoprecipitation was carried out as previously described (23), with the exception that EDTA was excluded, and 250 units/mL benzonase (Merck) and 1.5 mM MgCl_2_ were added to the lysis buffer. Anti-RECQL1 antibody (5 μg, A300-450A) or a nonspecific IgG (Dako) coupled with protein A agarose beads (GE Healthcare) were used to isolate protein complexes from 5 mg whole-cell extract.

### Immunofluorescence microscopy.

Cells were seeded onto coverslips 24 hours before extraction and fixation. Cytochalasin B (2 μg/mL, MilliporeSigma) was added to the media for 24 hours to delete micronuclei. Cells were preextracted for 5 minutes on ice with ice-cold buffer (25 mM HEPES, pH 7.4, 50 mM NaCl, 1 mM EDTA, 3 mM MgCl_2_, 300 mM sucrose, and 0.5% Triton X-100) and then fixed with 4% paraformaldehyde for 10 minutes. Fixed cells were stained with primary antibodies against γ-H2AX (Merck, 05-636), RAD51 (Merck, PC130), 53BP1 (Novus Biologicals, NB100-904), RECQL1 (Abcam, ab151501), and mitosin (BD Transduction Laboratories, 610768), with secondary antibodies conjugated to Alexa Fluor 488 and Alexa Fluor 594 (Life Technologies, Thermo Fisher Scientific), and then with DAPI (VectaLabs). Images were visualized using a Nikon Eclipse Ni microscope with NIS-Elements software (Nikon Instruments) and captured using a 100× oil immersion objective.

### DNA fiber–spreading assay.

Cells were pulse-labeled with 25 μM CldU for 20 to 30 minutes, washed with PBS, pulse-labeled with 250 μM IdU with or without 50 nM CPT or 50 nM ETOP for 20 to 30 minutes, and then harvested. For replication restart experiments, cells were labeled with CldU, washed in warm PBS, and incubated in media containing 2 mM HU or 0.02% MMS for 2 hours or 20 minutes, respectively. Cells were washed again in warm PBS and then incubated with IdU for the indicated duration. DNA fiber analysis was carried out as previously described (45).

### Metaphase spreads.

Giemsa-stained metaphase spreads were prepared as previously described (45). Briefly, Colcemid (KaryoMAX, Thermo Fisher Scientific) was added at a final concentration of 0.2 μg/mL for 3 hours. Cells were then harvested by trypsinization, subjected to hypotonic shock for 30 minutes at 37°C in hypotonic buffer (10 mM KCl, 15% FCS), and fixed in 3:1 ethanol/acetic acid solution. Cells were dropped onto acetic acid–humidified slides, stained for 15 minutes in a Giemsa-modified solution (Merck; 5% vol/vol in water), and washed in water for 5 minutes before visualization by light microscopy.

### DNA helicase assays.

DNA helicase assays were performed as previously described (19). Briefly, reactions were carried out in a 20 μL volume with 20 mM Tris (pH 7.4), 10 mM KCl, 5 mM ATP, 5 mM MgCl_2_, 10% glycerol, and 80 ng/μL BSA for 15 minutes at 37°C. Reaction mixtures containing a forked duplex DNA substrate (19 bp or 31 bp) were incubated with the indicated concentrations of WT RECQL1 or RECQL1-A459S helicase proteins. Reactions were quenched with an equal volume of 2× stop dye (18 mM EDTA, 0.6% SDS, 25% glycerol, 0.4% bromophenol blue, 0.4% xylene cyanol, and 0.1 mg/mL proteinase K) and incubated for an additional 15 minutes at 37°C to remove bound RECQL1 protein. Reaction products were resolved by electrophoresis on native 12% polyacrylamide gels at 200 V for 1.5 hours. Radiolabeled DNA products were visualized with a phosphoimager and quantified using ImageQuant software (Cytiva). For kinetics analysis of DNA unwinding, 19 bp or 31 bp forked duplex DNA substrates were incubated with the specified RECQL1 concentration and incubated for the indicated durations at 37°C. The rates of helicase-catalyzed DNA unwinding were determined by linear regression analysis.

### Strand annealing assays.

Strand annealing assays were performed as previously described (19). Briefly, the specified concentration of WT RECQL1 or RECQL1-A459S helicase protein was incubated with 0.5 nM each of 5′-end radiolabeled oligonucleotide and partially complementary unlabeled oligonucleotide. Strand annealing reactions (20 μL) were carried out in helicase reaction buffer (20 mM Tris-HCl [pH 7.5], 10 mM KCl, 8 mM dithiothreitol, 5 mM MgCl_2_) and quenched by addition of an equal volume of 2× stop dye (18 mM EDTA, 0.6% SDS, 25% glycerol, 0.4% bromophenol blue, 0.4% xylene cyanol and 0.1 mg/mL proteinase K) and incubated for an additional 15 minutes at 37 °C to remove bound RECQL1 protein. Reaction products were electrophoresed on native 12% polyacrylamide gels at 200 V for 1.5 hours. Radiolabeled products were visualized and quantified as described above.

### Fork restoration kinetics assay.

WT or A459S RECQL1 (20 nM) was incubated with 2 nM radiolabeled reversed fork substrate, 2 mM ATP, 15 mM phosphocreatine, and 30 u/mL creatine phosphokinase in reaction salts (35 mM Tris-HCl, pH 7.5, 20 mM KCl, 5 mM MgCl_2_, 0.1 mg/mL BSA, 2 mM DTT, 5% glycerol) in 100 μL reactions at 37°C. Aliquots of 20 μL were removed at the indicated times and were quenched by the addition of 10 μL 3X stop buffer (1.2% SDS, 30% glycerol and proteinase K), followed by incubation for at least 10 minutes at room temperature. Separate reactions containing either a reversed fork and replication fork structure without RECQL1 were incubated for 30 minutes at 37 °C followed by addition of the 3× stop buffer. Samples were immediately loaded onto an 8% polyacrylamide 1× TBE gel and electrophoresed for 4 hours at 200 V and 4°C. Gels were exposed to a phosphoimager screen overnight and then imaged using a Typhoon FLA 9500 imager (Cytiva). Gel images were quantified using ImageQuant TL software (Cytiva), and raw data were processed using Microsoft Excel. The percentage fork restoration was calculated by dividing the volume of the replication fork band by the total volume at each time point and subtracting the percentage of the fork restored in the no-enzyme control from each time point. Data representing 3 replicates of the assay were averaged and graphed.

### Study approval.

Written informed consent to publish clinical information and photographs of the affected individuals was obtained from the families prior to their involvement in this study, in accordance with the IRB-approved protocol 0306-10-HMO from the Hadassah Medical Center (Jerusalem, Israel). Further approval for this research was obtained from the West Midlands, Coventry, and Warwickshire Research Ethics Committee (Coventry, United Kingdom; REC: 20/WM/0098).

## Author contributions

TH, VMP, AAL, and BAL identified the patients with the RECQL1 mutation and provided clinical information and photographs of the affected individuals. TH and VM analyzed the whole-exome sequencing data. KDB performed segregation analysis and established patient-derived LCLs. SSJ, SLC, and AD performed DNA fiber analysis. LJG, JJR, and RH performed flow cytometric, SCE, and chromosome breakage analyses. BLW performed growth and cytotoxicity assays on LCLs. GSM performed chromatin fractionation and immunofluorescence. SJS performed structural modeling. CNM and GLM generated the RECQL1 CRISPR-KO HeLa cell line. GSS generated complemented cell lines, carried out immunofluorescence and co-immunoprecipitation studies, and performed Western blotting. SD and JAS purified recombinant RECQL1 and performed in vitro protein biochemical assays. SD and AD performed cell-based DNA damage sensitivity assays and the comet assay. ANG generated RECQL1 expression vectors. GMCL and VR carried out C-Fusion 3D high-throughput imaging. GSS, SSJ, and RMB planned and supervised the study and wrote the manuscript.

## Supplementary Material

Supplemental data

## Figures and Tables

**Table 2:**
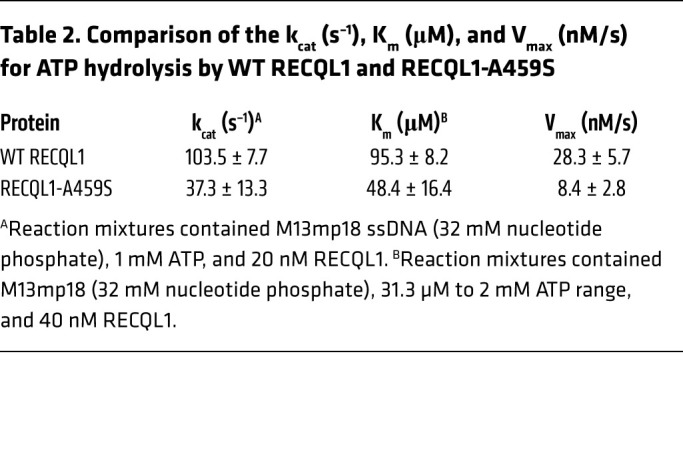
Comparison of the k_cat_ (s^–1^), K_m_ (μM), and V_max_ (nM/s) for ATP hydrolysis by WT RECQL1 and RECQL1-A459S

**Table 1:**
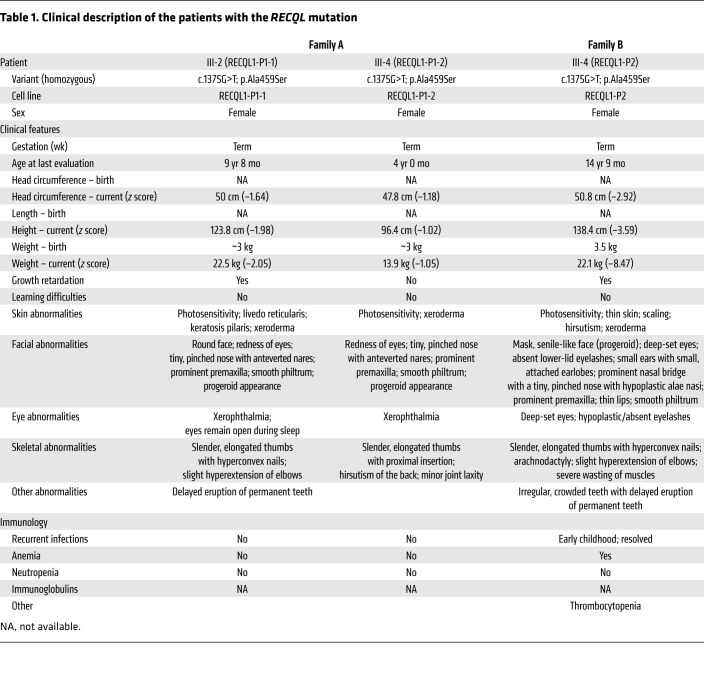
Clinical description of the patients with the *RECQL* mutation

## Version 1 01/13/2022

In-Press Preview

## Version 2 03/01/2022

Electronic publication
