# Supplementary material for: RECON syndrome is a genome instability disorder caused by mutations in the DNA helicase RECQL1
Source: J Clin Invest. 2022 Mar 1;132(5):e147301. doi: 10.1172/JCI147301 (PMC8884905; doi:10.1172/JCI147301)
Supplement: Supplemental data [file jci-132-147301-s176.pdf]

Supp. Fig. 1

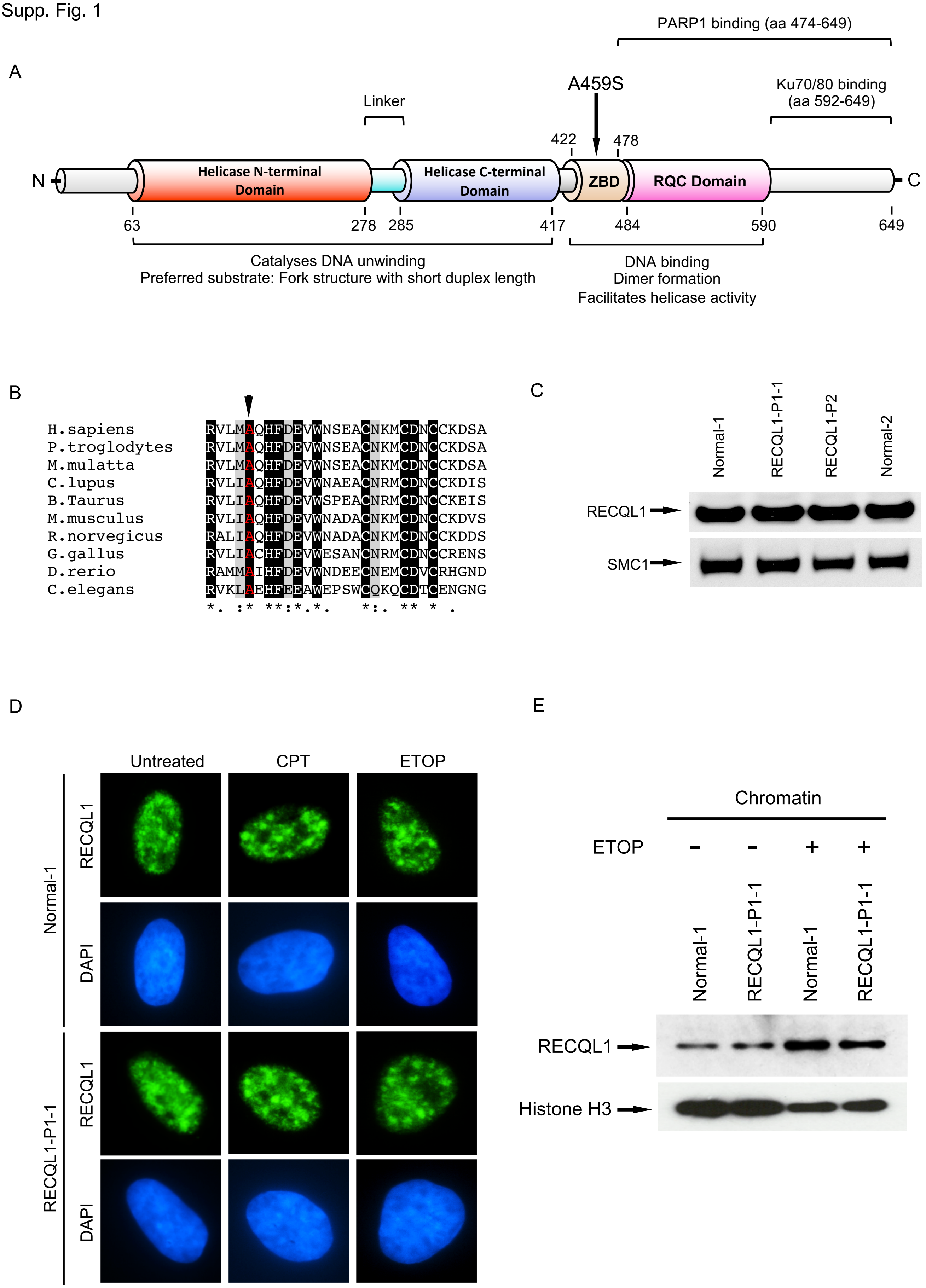
A
PARP1 binding (aa 474-649)
A459S
Ku70/80 binding
(aa 592-649)
Linker
422
478
N
Helicase N-terminal Domain
Helicase C-terminal Domain
ZBD
RQC Domain
C
63
278
285
417
484
590
649
Catalyses DNA unwinding
Preferred substrate: Fork structure with short duplex length
DNA binding
Dimer formation
Facilitates helicase activity
B
H.sapiens RVLMAQHFDEVWNSEACNKMCDNCCKDSA
P.troglodytes RVLMAQHFDEVWNSEACNKMCDNCCKDSA
M.mulatta RVLMAQHFDEVWNSEACNKMCDNCCKDSA
C.lupus RVLIAQHFDEVWNAEACNRMCDNCCKDIS
B.Taurus RVLIAQHFDEVWSPEACNRMCDNCCKEIS
M.musculus RVLIAQHFDEVWNADACNKMCDNCCKDVS
R.norvegicus RALIAQHFDEVWNADACNKMCDNCCKDDS
G.gallus RVLIACHFDEVWESANCNRMCDNCCRENS
D.rerio RAMMAIHFDEVWNDEECNEMCDVCRHGND
C.elegans RVKLAEHFEEAWEPSWCQKQCDTCENGNG
. : **:*.*. *:. ** * .
C
Normal-1
RECQL1-P1-1
RECQL1-P2
Normal-2
RECQL1
SMC1
D
Untreated
CPT
ETOP
Normal-1
RECQL1
DAPI
RECQL1-P1-1
RECQL1
DAPI
E
Chromatin
ETOP
-
-
+
+
Normal-1
RECQL1-P1-1
Normal-1
RECQL1-P1-1
RECQL1
Histone H3

Supp. Fig. 1

F

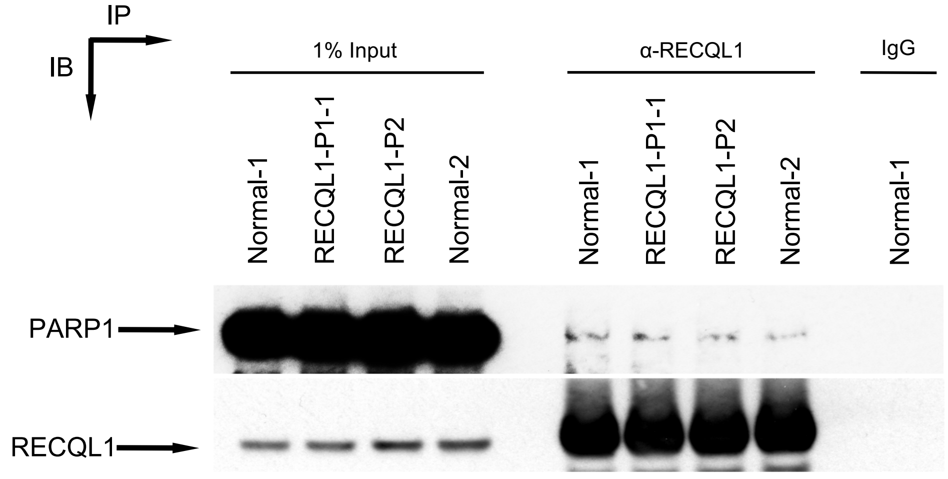

IP
IB
1% Input
α-RECQL1
IgG
Normal-1
RECQL1-P1-1
RECQL1-P2
Normal-2
Normal-1
RECQL1-P1-1
RECQL1-P2
Normal-2
Normal-1
PARP1
RECQL1

Supplementary Figure 1. Identification of a homozygous mutation in *RECQL* (c.G1375T; p.A459S) in the affected individuals from family A and B. (**A**) A schematic of the RECQL1 protein with the amino acid position of known domains shown. The position of the p.A459S mutation is indicated with an arrow. Functional domains and sites of protein binding are indicated. (**B**) Multiple sequence alignment of RECQL1 protein across the position of amino acid alanine-459 from different species. The position of alanine-459 is highlighted in red and marked by an arrow. (**C**) Western blot of whole cell extracts from two normal lymphoblastoid cell lines (LCLs) (normal-1 and normal-2) and LCLs from two of the *RECQL* mutant patients (patient III-2, family A – RECQL1-P1-1; patient III-4, family B – RECQL1-P2) using antibodies to RECQL1 and SMC1. SMC1 was used as a protein loading control. The RECQL1 and SMC1 Western blots were generated from cutting the same nitrocellulose filter into strips and blotting with the antibodies indicated. (**D**) Representative immunofluorescence images showing the cellular localisation of WT and p.A459S mutant RECQL1 in normal and RECQL1-P1-1 patient-derived fibroblasts before and after exposure to CPT and ETOP. (**E**) Western blot of fractionated normal and RECQL1-P1-1 patient-derived fibroblasts assessing the chromatin localisation of WT and p.A459S mutant RECQL1 before and after exposure to ETOP. The RECQL1 and Histone H3 Western blots were generated from cutting the same nitrocellulose filter into strips and blotting with the antibodies indicated. (**F**) Co-immunoprecipitation analysis using normal and patient-derived LCLs examining the impact of the p.A459S RECQL1 mutation on the association of RECQL1 with PARP1 in the absence of exogenous DNA damage. IP: immunoprecipitation; IB: Immunoblot. The antibodies used for the immunoprecipitation and Western blot are indicated. IgG indicates a isotype matched IgG used for immunoprecipitation to control for non-specific protein binding to immunoglobulins.

Supp. Fig. 2

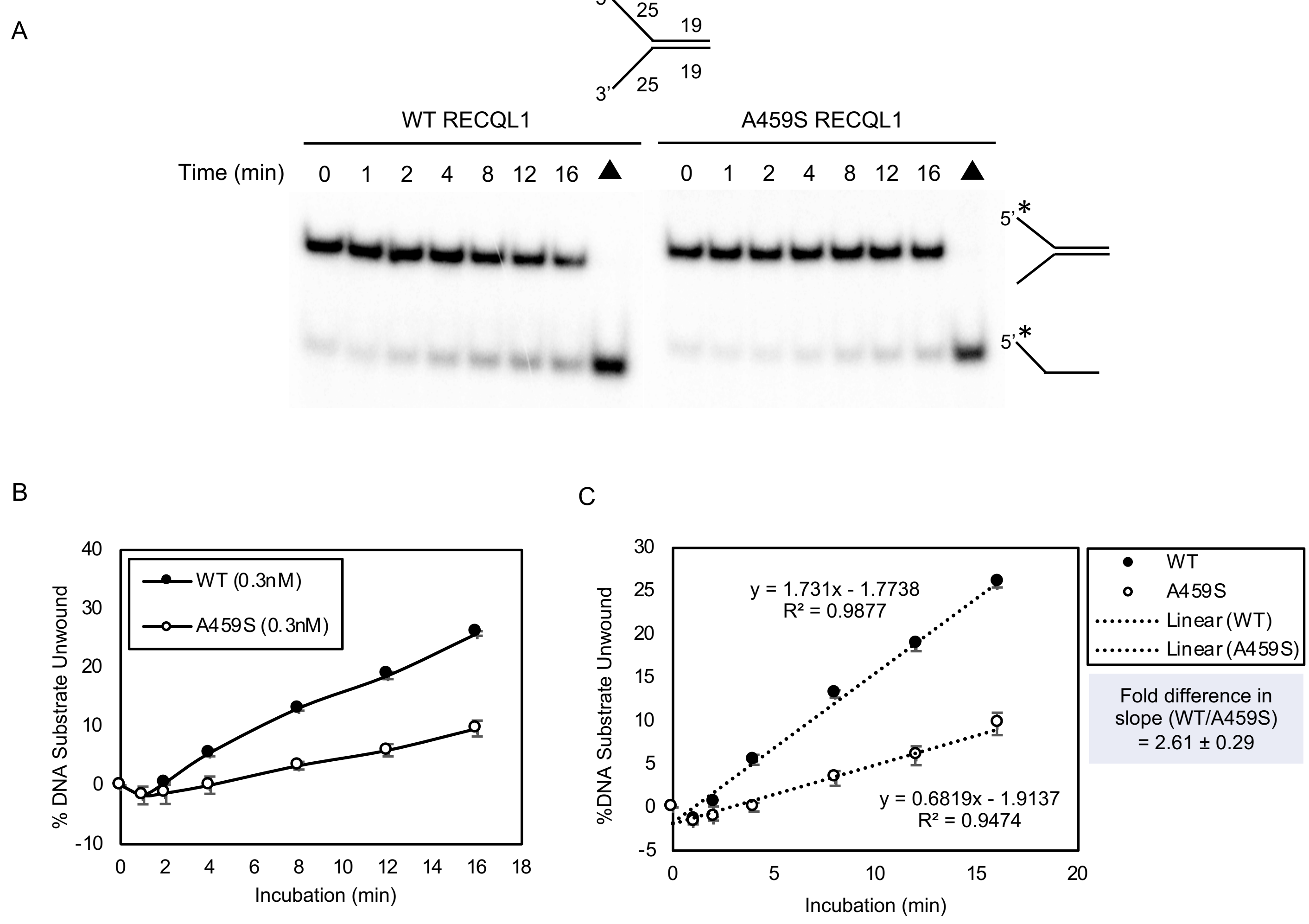
A
5'*
25
19
3'
25
19
WT RECQL1
A459S RECQL1
Time (min)
0 1 2 4 8 12 16
0 1 2 4 8 12 16
5'*
5'*
B
WT (0.3nM)
A459S (0.3nM)
% DNA Substrate Unwound
Incubation (min)
40
30
20
10
0
-10
0 2 4 6 8 10 12 14 16 18
C
%DNA Substrate Unwound
Incubation (min)
y = 1.731x - 1.7738
R² = 0.9877
y = 0.6819x - 1.9137
R² = 0.9474
30
25
20
15
10
5
0
-5
0 5 10 15 20
WT
A459S
Linear (WT)
Linear (A459S)
Fold difference in slope (WT/A459S) = 2.61 ± 0.29

Supplementary Figure 2. The p.A459S mutation compromises the DNA unwinding activity of RECQL1. (**A**) Kinetic analyses of DNA unwinding by RECQL1-WT or RECQL1-A459S. Representative gel images showing analysis of reaction mixtures containing 0.3 nM RECQL1-WT or RECQL1-A459S helicase incubated with a 19 bp forked duplex DNA substrate for the indicated times. The black triangle indicates the unduplexed radiolabelled oligonucleotide, which is used as a marker for duplex unwinding. (**B**) Quantitation of DNA unwinding from three independent experiments with SEM. Area under the curve (AUC): WT (mean±SEM: 197.2±4.521); A459S (mean±SEM: 60.26±8.332). WT vs A459S AUC unpaired t-test: $p<0.0001$. (**C**) Quantitation of DNA unwinding showing a linear trendline and slope of unwinding from three independent experiments with SEM.

Supp. Fig. 3

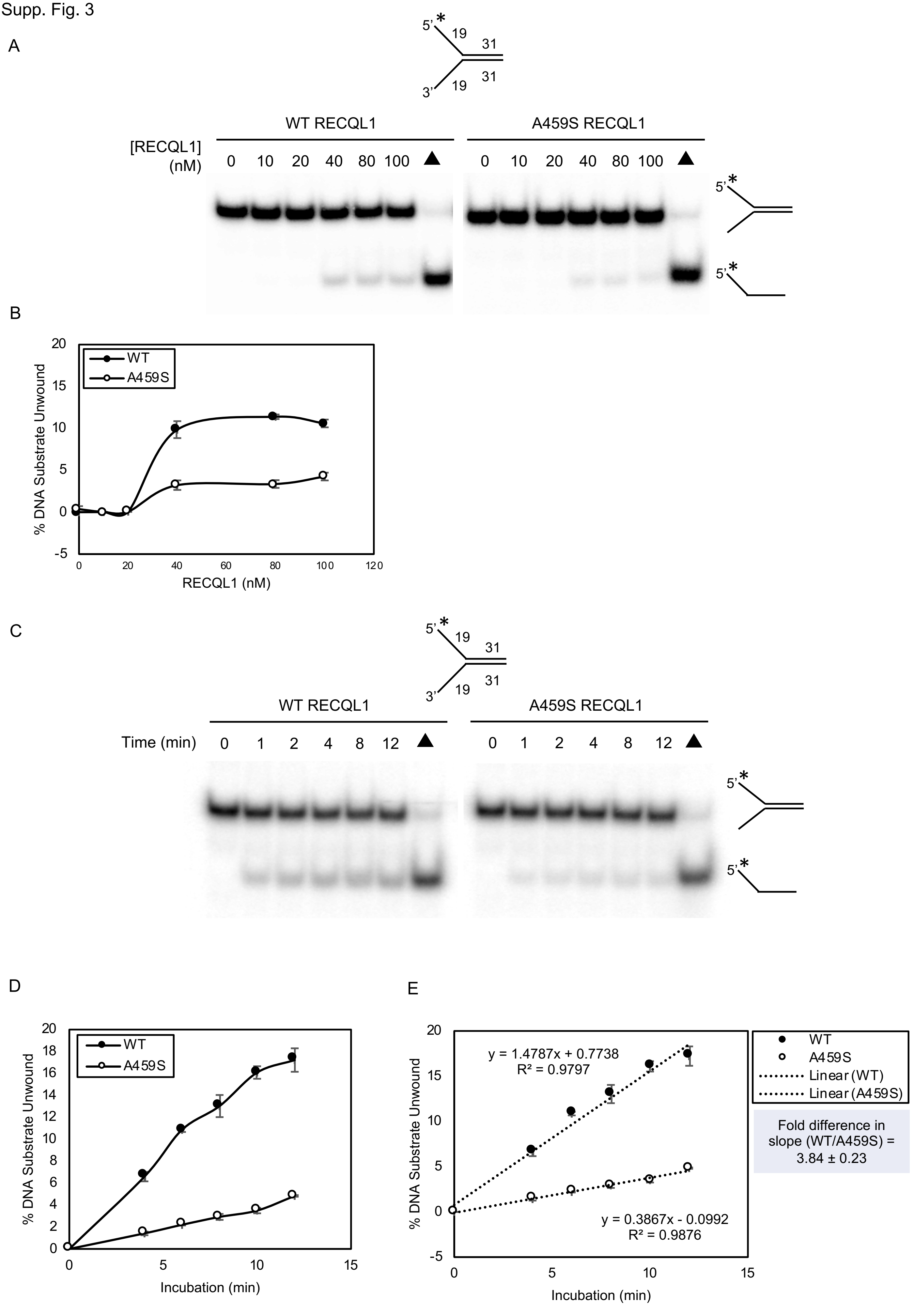
A
5'*
19
31
3'
19
31
WT RECQL1
A459S RECQL1
[RECQL1] (nM)
0 10 20 40 80 100
0 10 20 40 80 100
B
WT
A459S
% DNA Substrate Unwound
RECQL1 (nM)
C
Time (min)
0 1 2 4 8 12
0 1 2 4 8 12
D
% DNA Substrate Unwound
Incubation (min)
E
y = 1.4787x + 0.7738
R² = 0.9797
y = 0.3867x - 0.0992
R² = 0.9876
Linear (WT)
Linear (A459S)
Fold difference in slope (WT/A459S) = 3.84 ± 0.23
Incubation (min)

Supp. Fig. 3

F

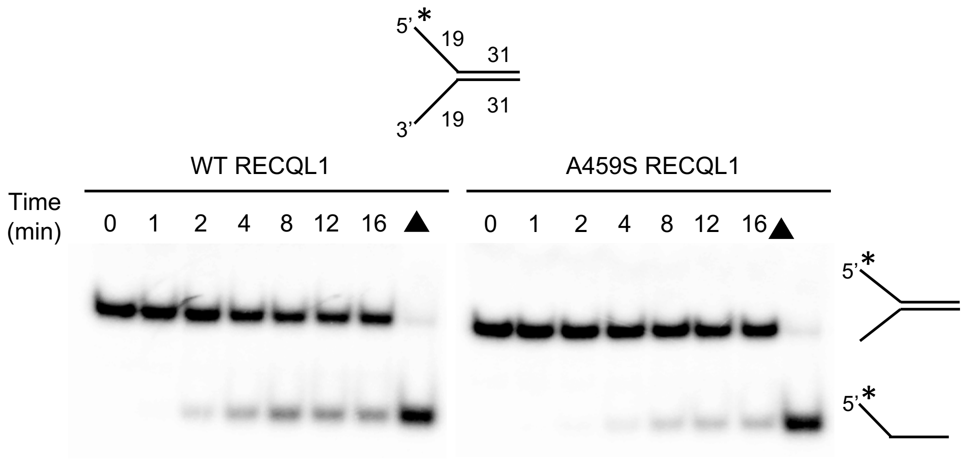

G

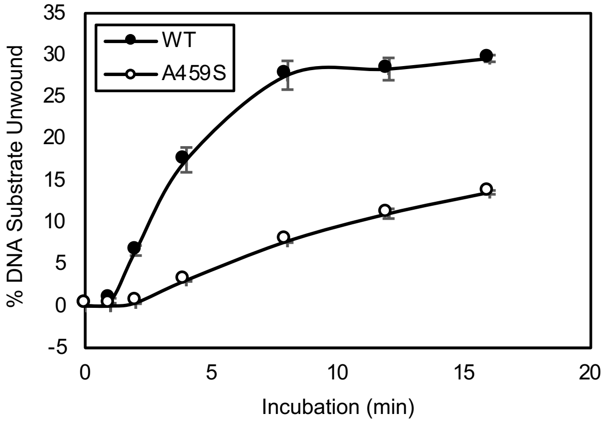

H

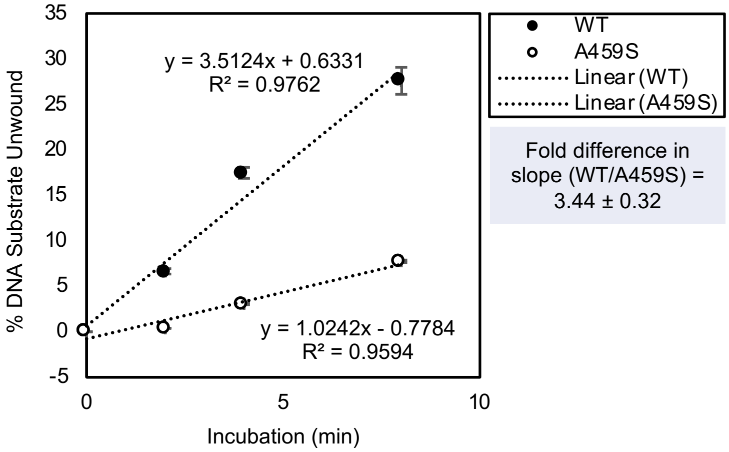

Supplementary Figure 3. RECQL1-A459S is compromised in its DNA unwinding activity as analyzed utilizing a 31-bp forked duplex DNA substrate. (**A**) Representative gels showing analysis of reaction mixtures containing RECQL1-WT or RECQL1-A459S incubated with a 31 bp forked DNA substrate for 15 min. (**B**) Quantitation of DNA unwinding from three independent experiments with SEM. Area under the curve (AUC): WT (mean±SEM: 744.6±41.26); A459S (mean±SEM: 243.5±29.61). WT vs A459S AUC unpaired t-test: $p<0.0453$. (**C**) Kinetic analyses of DNA unwinding by RECQL1-WT or RECQL1-A459S. Representative gels showing analysis of reaction mixtures containing 30 nM RECQL1-WT or RECQL1-A459S incubated with a 31 bp forked DNA substrate for the indicated times. (**D**) Quantitation of DNA unwinding. AUC: WT (mean±SEM: 103.7±3.508); A459S (mean±SEM: 23.49±0.9705). WT vs A459S AUC unpaired t-test: $p<0.0001$. (**E**) linear trendline and slope of three independent experiments with SEM. (**F**) Representative gels showing analysis of reaction mixtures containing 40 nM RECQL1-WT or RECQL1-A459S incubated with a 31 bp forked DNA substrate for the indicated times. (**G**) Quantitation of DNA unwinding. AUC: WT (mean±SEM: 345.6±14.99); A459S (mean±SEM: 112.2±3.837). WT vs A459S AUC unpaired t-test: $p<0.0001$. (**H**) linear trendline and slope of three independent experiments with SEM.

Supp. Fig. 4

A

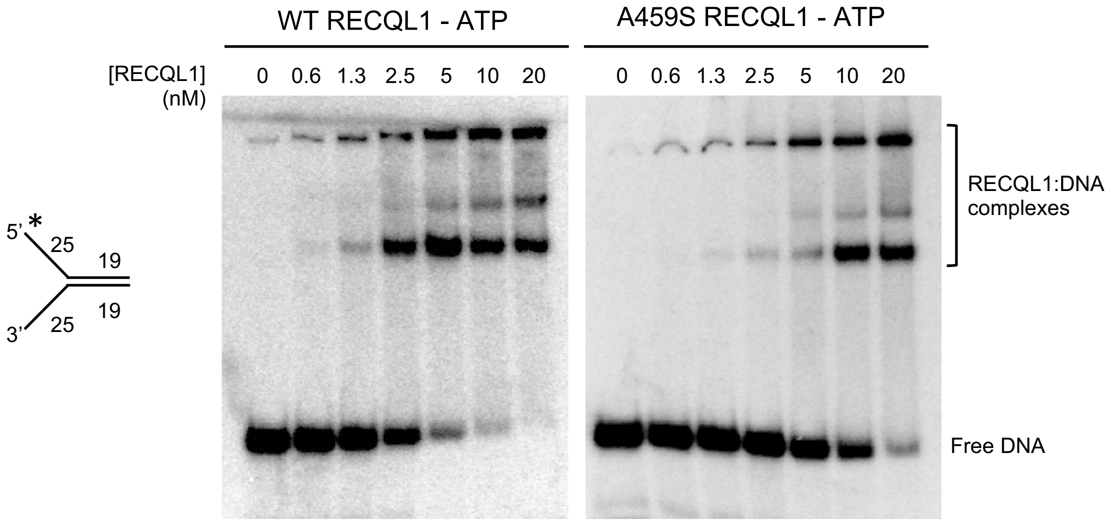

K_d values

WT	A459S
2.94 ± 0.14	4.74 ± 0.41

B

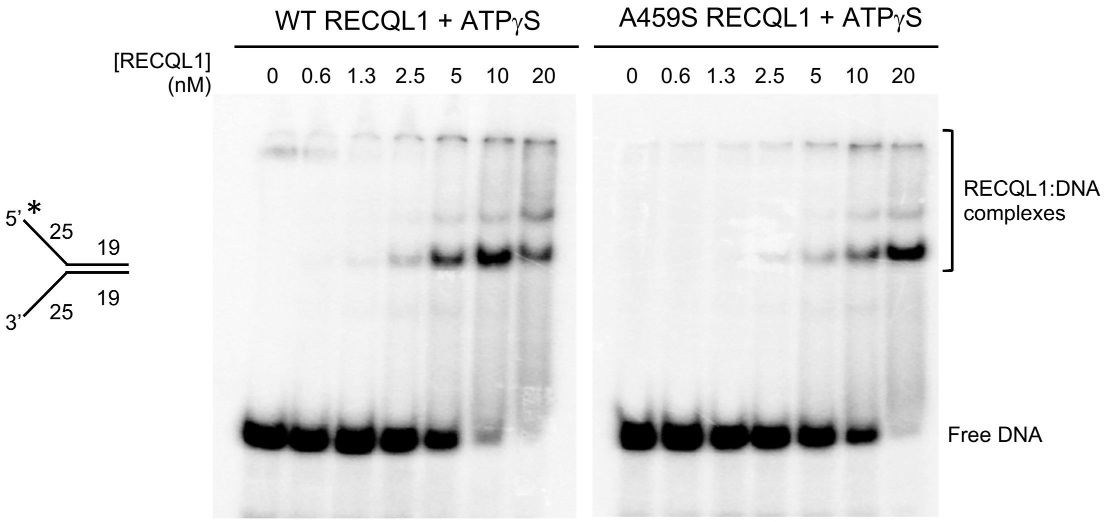

K_d values

WT + ATPγS	A459S + ATPγS
5.39 ± 0.70	7.69 ± 0.62

Supplementary Figure 4. The p.A459S mutation has a mild impact on the ability of RECQL1 to bind DNA. Representative gel images showing analysis of DNA binding mixtures containing RECQL1-WT or RECQL1-A459S incubated with a 19 bp forked DNA substrate. RECQL1-WT or RECQL1-A459S (0.3 – 20 nM) were incubated with a 19 bp forked duplex DNA substrate in the absence (**A**) or presence (**B**) of ATPγS. Average K_d values determined from three independent experiments with SEM are shown.

Supp. Fig. 5

A

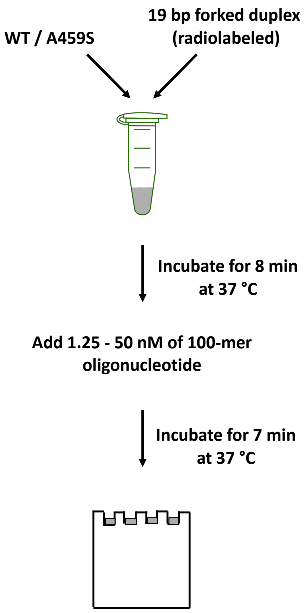

B

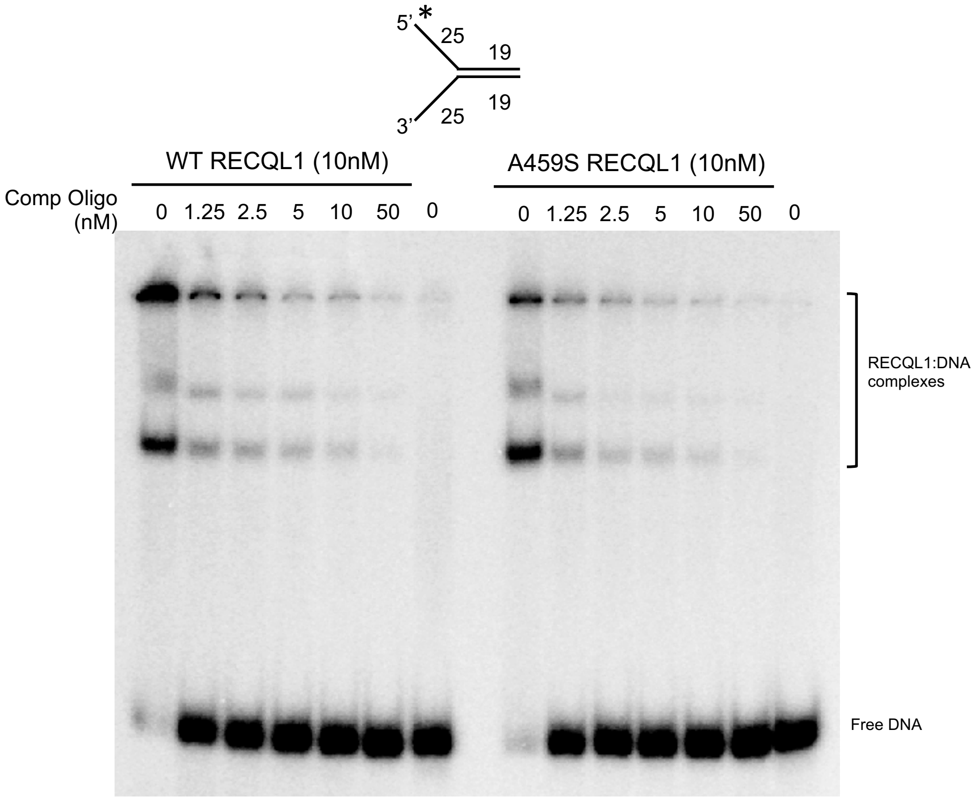

C

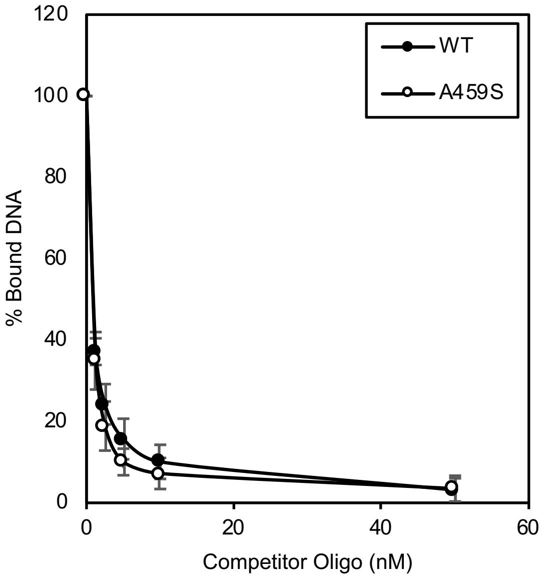

Supplementary Figure 5. The p.A459S mutation does not affect the stability of pre-formed forked duplex-RECQL1 protein complexes. (**A**) Diagrammatic representation of the experimental protocol. (**B**) Representative gel images showing analysis of DNA binding mixtures from experiments with unlabelled competitor oligonucleotide. RECQL1-WT or RECQL1-A459S (10 nM) were pre-incubated with 0.5 nM radiolabelled 19-bp forked duplex DNA substrate in the absence or presence of increasing concentrations of unlabelled competitor oligonucleotide ranging from 1.25 - 50 nM, as described in Materials and Methods. (**C**) Quantitation of six independent experiments with SEM. Data were normalized to the percentage of DNA bound by RECQL1-WT or RECQL1-A459S in the absence of competitor oligonucleotide.

Supp. Fig. 6

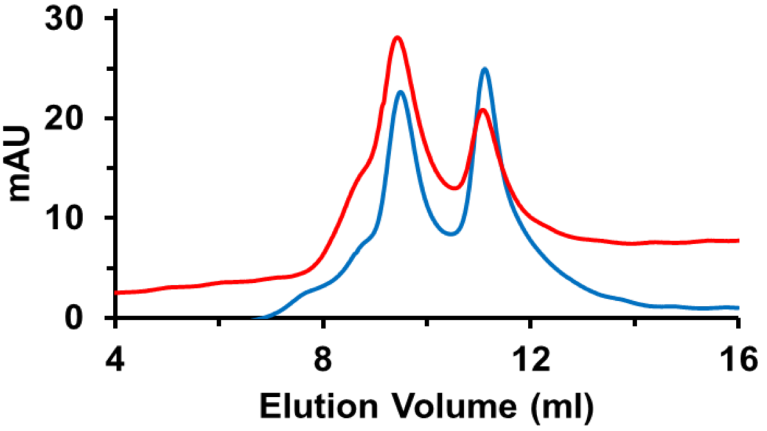

RECQL1-WT (red) and RECQL1-A459S (blue)

Supplementary Figure 6. RECQL1-A459S protein retains its ability to oligomerize. Chromatogram from a size exclusion column showing the distribution of purified RECQL1-WT (red) and RECQL1-A459S (blue) proteins in the absence of ATP. Both RECQ1-WT and RECQL1-A459S show two distinct peaks of elution at 9.5 ml and 11.1 ml.

Supp. Fig. 7

A

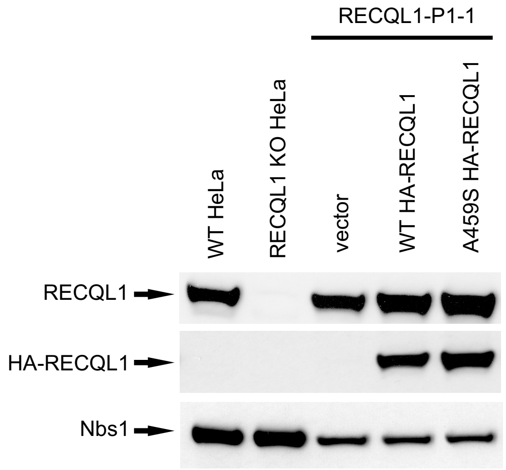
RECQL1-P1-1
WT HeLa
RECQL1 KO HeLa
vector
WT HA-RECQL1
A459S HA-RECQL1
RECQL1
HA-RECQL1
Nbs1

B

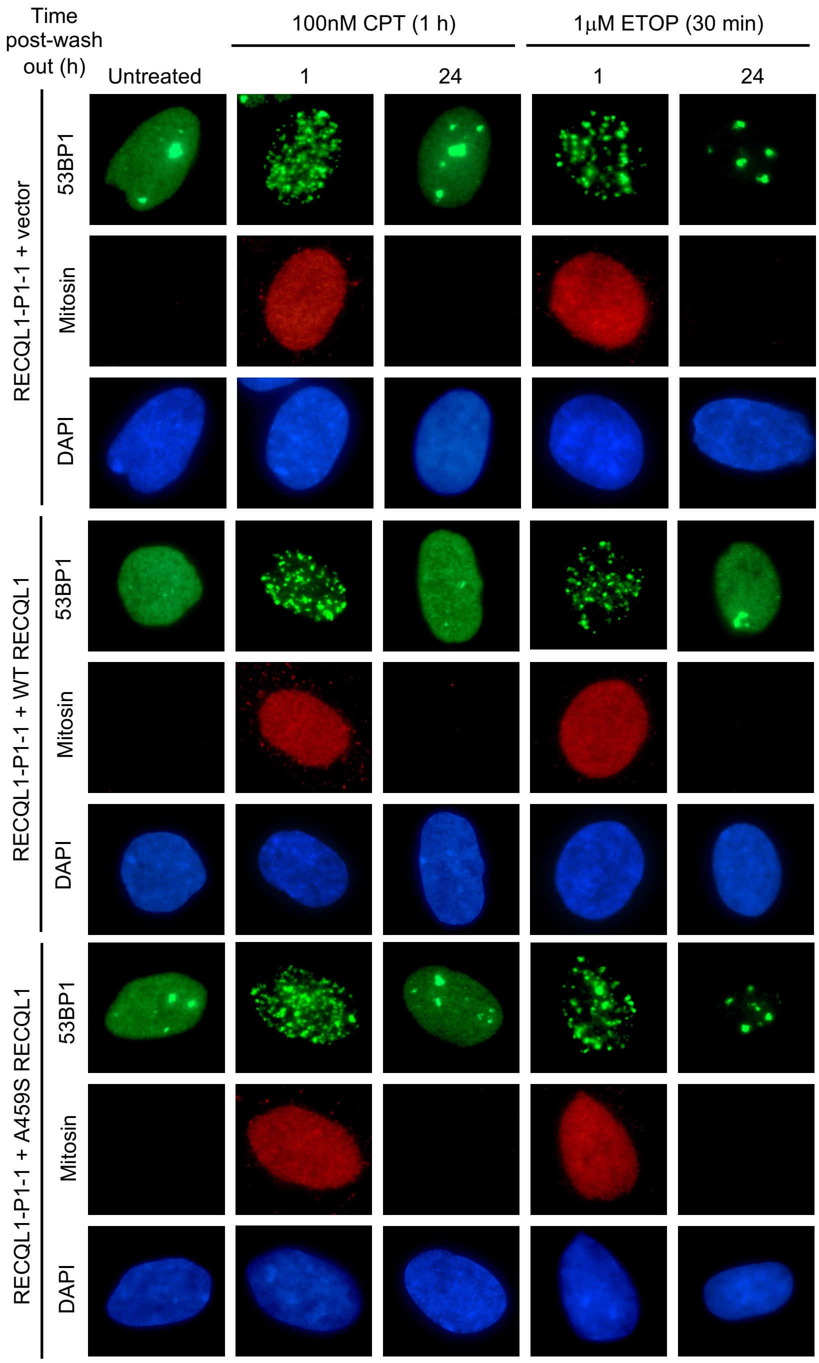
Time post-wash out (h)
100nM CPT (1 h)
1μM ETOP (30 min)
Untreated
1
24
1
24
RECQL1-P1-1 + vector
RECQL1-P1-1 + WT RECQL1
RECQL1-P1-1 + A459S RECQL1
53BP1
Mitosin
DAPI

Supp. Fig. 7

C

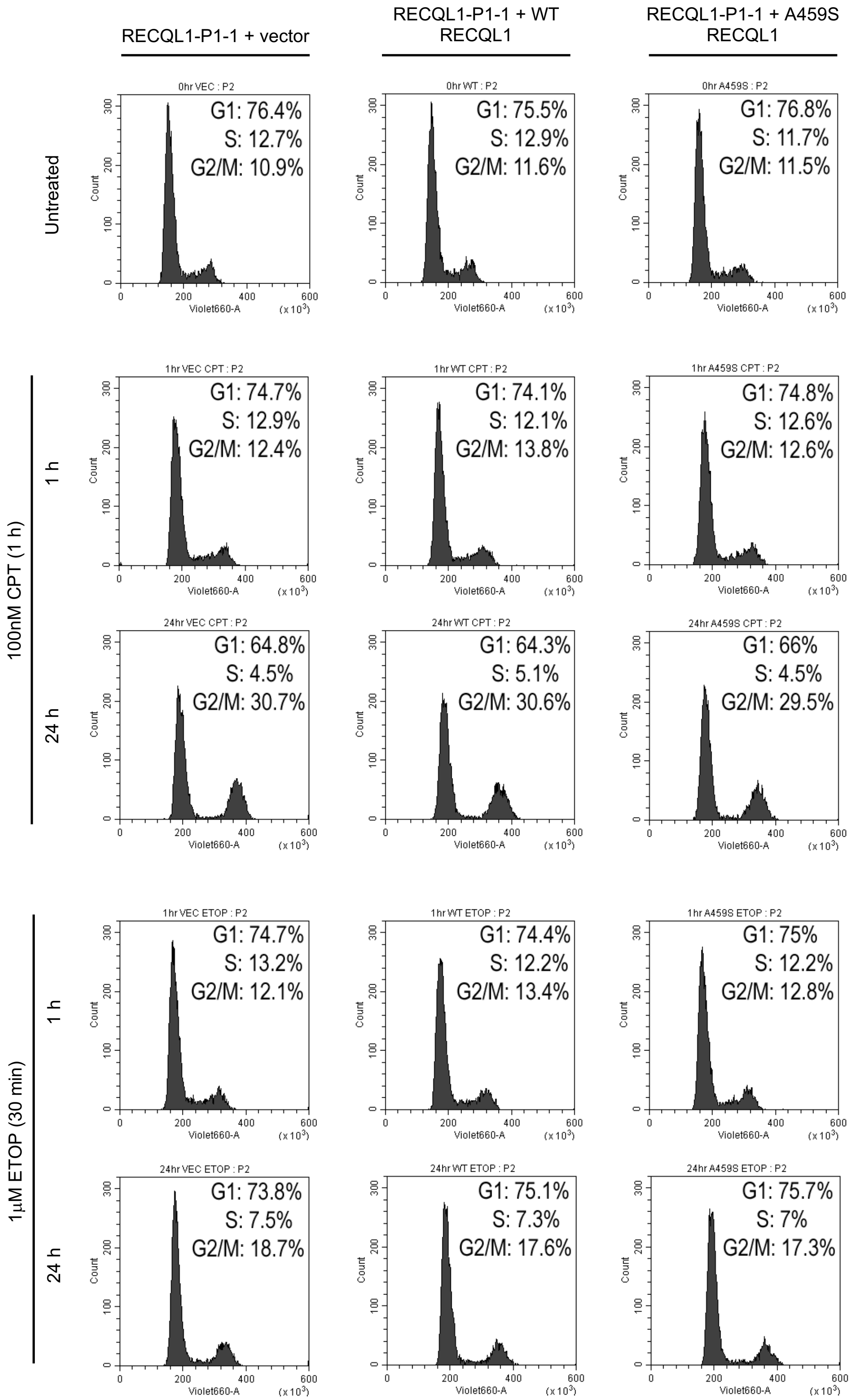
RECQL1-P1-1 + vector
RECQL1-P1-1 + WT RECQL1
RECQL1-P1-1 + A459S RECQL1
Untreated
100nM CPT (1 h)
1 h
24 h
1μM ETOP (30 min)
1 h
24 h
0hr VEC : P2
G1: 76.4%
S: 12.7%
G2/M: 10.9%
0hr WT : P2
G1: 75.5%
S: 12.9%
G2/M: 11.6%
0hr A459S : P2
G1: 76.8%
S: 11.7%
G2/M: 11.5%
1hr VEC CPT : P2
G1: 74.7%
S: 12.9%
G2/M: 12.4%
1hr WT CPT : P2
G1: 74.1%
S: 12.1%
G2/M: 13.8%
1hr A459S CPT : P2
G1: 74.8%
S: 12.6%
G2/M: 12.6%
24hr VEC CPT : P2
G1: 64.8%
S: 4.5%
G2/M: 30.7%
24hr WT CPT : P2
G1: 64.3%
S: 5.1%
G2/M: 30.6%
24hr A459S CPT : P2
G1: 66%
S: 4.5%
G2/M: 29.5%
1hr VEC ETOP : P2
G1: 74.7%
S: 13.2%
G2/M: 12.1%
1hr WT ETOP : P2
G1: 74.4%
S: 12.2%
G2/M: 13.4%
1hr A459S ETOP : P2
G1: 75%
S: 12.2%
G2/M: 12.8%
24hr VEC ETOP : P2
G1: 73.8%
S: 7.5%
G2/M: 18.7%
24hr WT ETOP : P2
G1: 75.1%
S: 7.3%
G2/M: 17.6%
24hr A459S ETOP : P2
G1: 75.7%
S: 7%
G2/M: 17.3%
Count
Violet660-A
(x 10³)

Supplementary Figure 7. Complementation of RECQL1-P1-1 fibroblasts with WT RECQL1 restores normal repair of DNA damaged induced by CPT and ETOP. (**A**) Western blot of cell extracts derived from wild type (WT) and RECQL1 CRISPR knockout HeLa cells and RECQL1-P1-1 fibroblasts complemented with empty vector, HA-tagged WT RECQL1 and RECQL1-A459S using antibodies to RECQL1, HA and NBS1 (used as a protein loading control). The RECQL1 and Nbs1 Western blots were generated from cutting the same nitrocellulose filter into strips and blotting with the antibodies indicated. The HA Western blot was generated by running the same samples in parallel on a separate gel, cutting the nitrocellulose filter into strips and blotting with the anti-HA antibody. (**B**) Representative immunofluorescent images showing the induction and repair of CTP and ETOP-induced DSBs using 53BP1 as a marker of DSBs and mitosin as a marker of S/G2 cells. DAPI is used to stain the nuclei of cells. Quantification of 53BP1 foci is shown in Figure 4A. (**C**) Cell cycle analysis of the cells used in (B) before and after exposure to CPT and ETOP using PI staining and flow cytometry.

Supp. Fig. 8

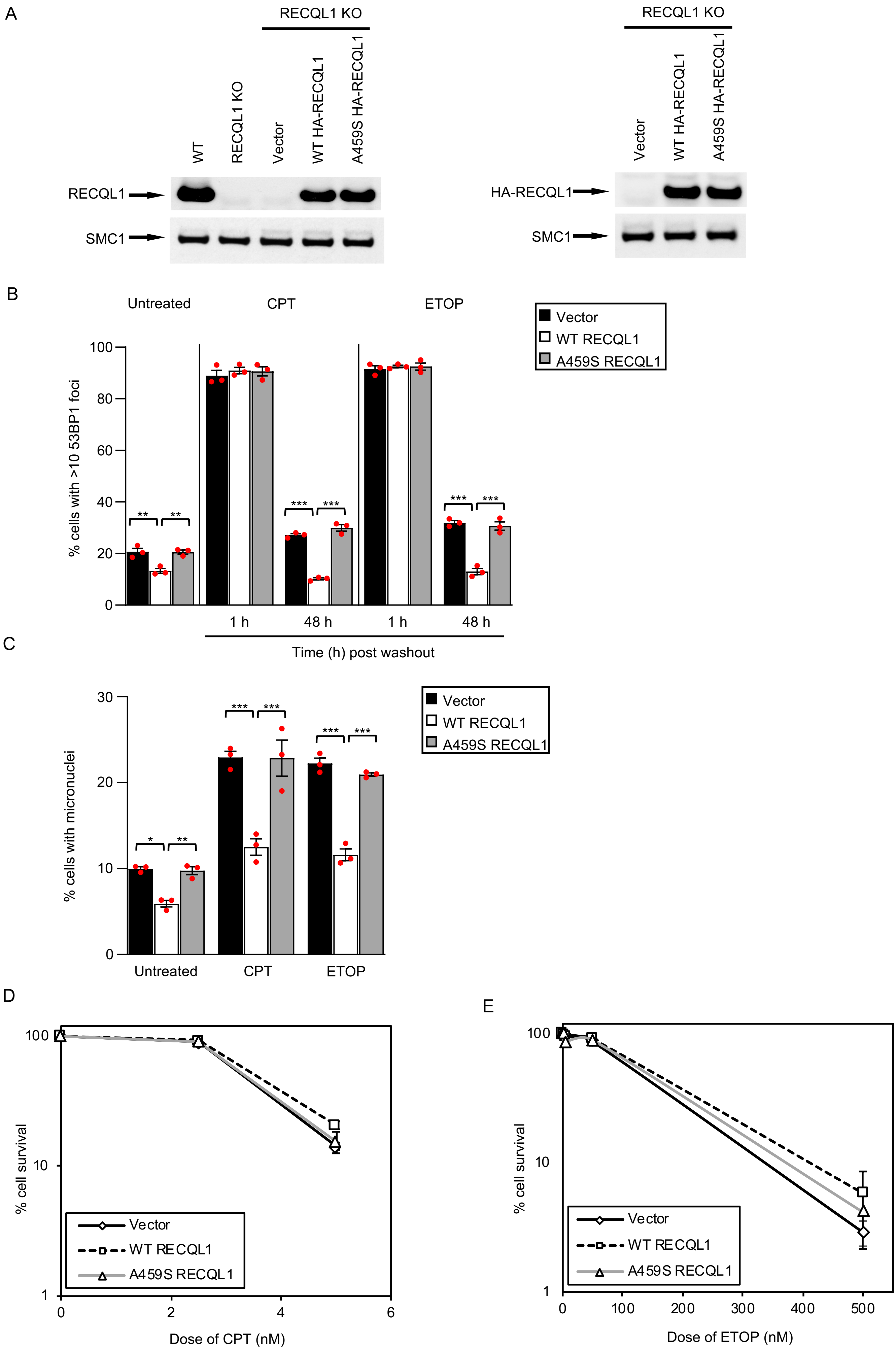
A
RECQL1 KO
WT
RECQL1 KO
Vector
WT HA-RECQL1
A459S HA-RECQL1
RECQL1
SMC1
RECQL1 KO
Vector
WT HA-RECQL1
A459S HA-RECQL1
HA-RECQL1
SMC1
B
Untreated
CPT
ETOP
Vector
WT RECQL1
A459S RECQL1
% cells with >10 53BP1 foci
100
80
60
40
20
0
**
**
***
***
***
***
1 h
48 h
1 h
48 h
Time (h) post washout
C
30
20
10
0
% cells with micronuclei
*
**
***
***
***
***
Vector
WT RECQL1
A459S RECQL1
Untreated
CPT
ETOP
D
100
10
1
% cell survival
Vector
WT RECQL1
A459S RECQL1
0
2
4
6
Dose of CPT (nM)
E
100
10
1
% cell survival
Vector
WT RECQL1
A459S RECQL1
0
100
200
300
400
500
Dose of ETOP (nM)

Supplementary Figure 8. Complementation of RECQL1 CRISPR knockout HeLa cells with mutant RECQL1 fails to restore normal repair of DNA damaged induced by exposure to CPT and ETOP. (**A**) Western blot of cell extracts derived from wild type (WT) HeLa cells, parental RECQL1 CRISPR knockout HeLa cells and RECQL1 CRISPR knockout HeLa cells complemented with empty vector, HA-tagged WT RECQL1 and RECQL1-A459S using antibodies to RECQL1, HA and SMC1. SMC1 was used as a protein loading control. The RECQL1 and SMC1 Western blots (left) were generated from cutting the same nitrocellulose filter into strips and blotting with the antibodies indicated. The HA and SMC1 Western blots (right) were generated by running the same samples in parallel on a separate gel, cutting the nitrocellulose filter into strips and blotting with the antibodies indicated. (**B**) Quantification of 53BP1 foci in complemented RECQL1 CRISPR knockout HeLa cells before and after (1 h and 48 h) treatment with 100 nM CPT (1 h) and 1 μM ETOP (30 min). 53BP1 foci in untreated cells and cells 24 h post-DNA damage induction were quantified in G1 phase only (mitosin negative). 53BP1 foci in cells 1 h post-DNA damage induction were quantified in S/G2 phase only (mitosin positive). The mean of three independent experiments is shown with the SEM. A minimum of 500 cells were counted per time point. Statistical significance was calculated using a one-way ANOVA with Tukey's multiple comparison. (**C**) Micronuclei were quantified from cells described in (B) before and 48 h after exposure to CPT and ETOP. The mean of three independent experiments is shown with the SEM. A minimum of 500 cells were counted per time point. Statistical significance was calculated using a two-way ANOVA with Dunnett's multiple comparison. (**D-E**) Colony survival analysis of the cells in (A) in response to varying dose of CPT (D) and ETOP (E). The data represents the mean of three independent experiments. (* p<0.05; ** p<0.01; ***p<0.001)

Supp. Fig. 9

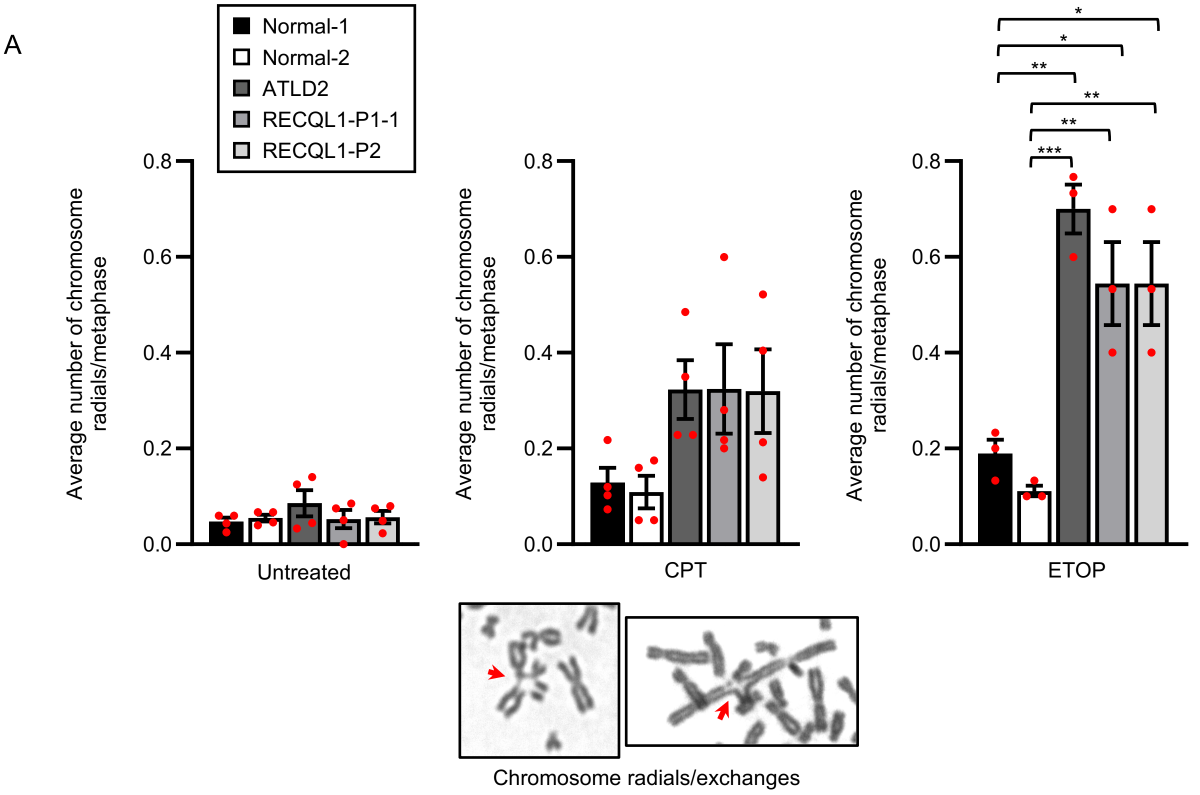
A
Normal-1
Normal-2
ATLD2
RECQL1-P1-1
RECQL1-P2
Average number of chromosome radials/metaphase
0.0
0.2
0.4
0.6
0.8
Untreated
CPT
ETOP
*
**
***
Chromosome radials/exchanges

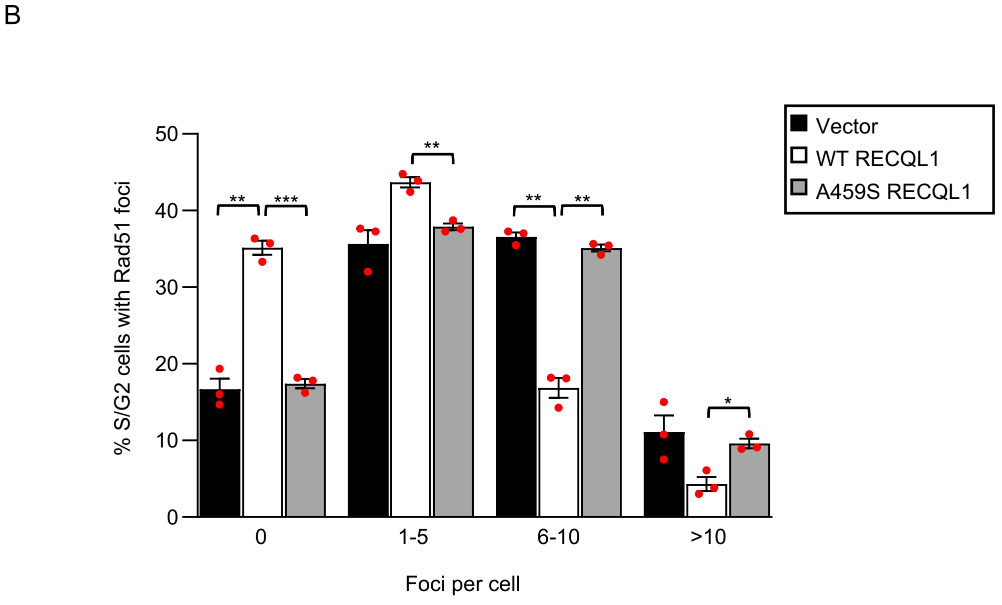
B
Vector
WT RECQL1
A459S RECQL1
% S/G2 cells with Rad51 foci
0
10
20
30
40
50
**
***
*
0
1-5
6-10
>10
Foci per cell

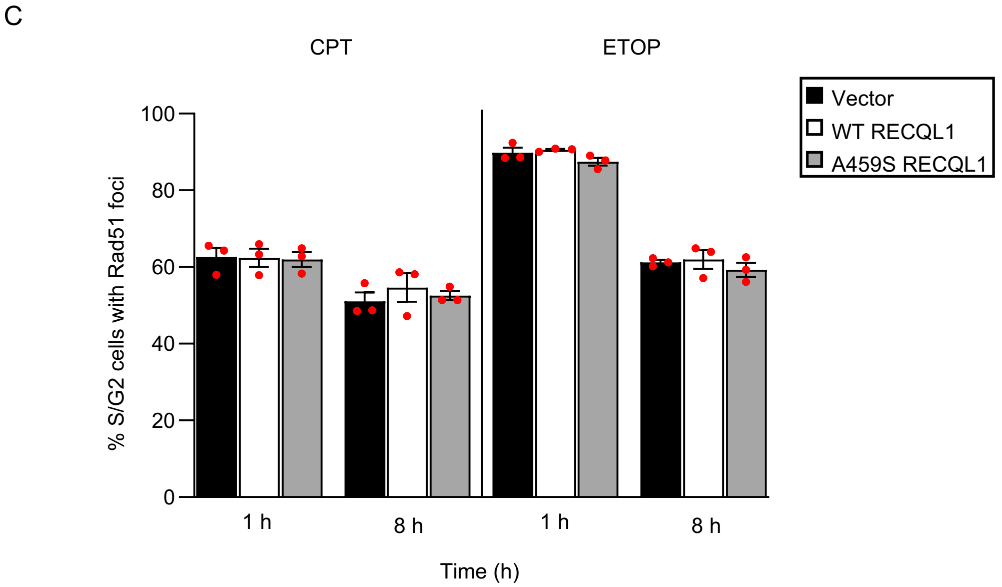
C
CPT
ETOP
Vector
WT RECQL1
A459S RECQL1
% S/G2 cells with Rad51 foci
0
20
40
60
80
100
1 h
8 h
1 h
8 h
Time (h)

Supp. Fig. 9

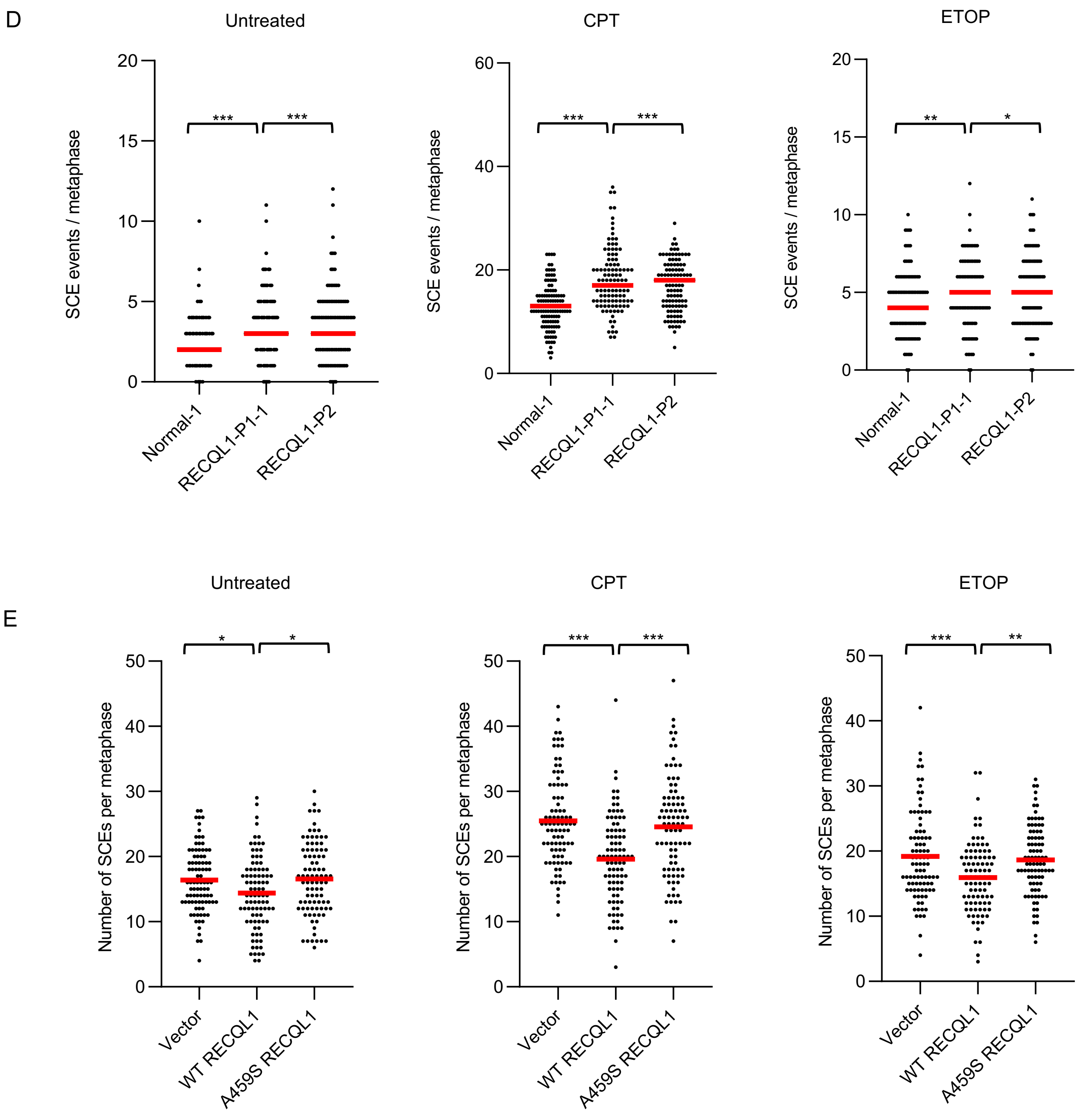
D
Untreated
CPT
ETOP
SCE events / metaphase
***
**
*
Normal-1
RECQL1-P1-1
RECQL1-P2
E
Number of SCEs per metaphase
Vector
WT RECQL1
A459S RECQL1

Supplementary Figure 9. The p.A459S RECQL1 mutation gives rise to a defect in the repair of abortive Top1/2 lesions but does not affect DNA damage induced Rad51 foci formation. (**A**) Quantification of chromosome radials/exchanges as a marker of defective HR in two normal LCLs, two RECQL mutant LCLs and an ATLD LCL before and 24 h after chronic exposure to low dose CPT (5 nM) and ETOP (50 nM). Representative images of a chromosome radial/exchange are shown. The mean of three independent experiments is shown with the SEM. A minimum of 50 metaphases were counted per cell line in each experiment. Statistical significance was calculated using a one-way ANOVA with Tukey's multiple comparison. (**B-C**) Quantification of Rad51 foci in complemented RECQL1-P1-1 patient fibroblasts before (B) and after treatment with 100 nM CPT (1 h) and 10 μM ETOP (30 min) (C). 1 h and 8 h timepoints following removal of CPT and ETOP from the cells were quantified. Rad51 foci were quantified in S/G2 phase only (mitosin positive). The mean of three independent experiments is shown with the SEM. A minimum of 500 cells were counted per time point. Statistical significance was calculated using a two-way ANOVA with Dunnett's multiple comparison. (**D**) Quantification SCEs in RECON LCLs before and 24 h after chronic exposure to low dose CPT (0.5 nM) and ETOP (10 nM). The median of three independent experiments is shown (red line). Approximately 40 metaphases were scored for SCEs per experiment. Statistical significance was calculated using a Kruskal-Wallis test with Dunn's multiple comparison. (**E**) Quantification of SCEs in complemented RECQL1 CRISPR knockout HeLa cells before and after exposure to CPT and ETOP (0.5 nM) and ETOP (10 nM). The median of three independent experiments is shown (red line). Approximately 30 metaphases were scored for SCEs per experiment. Statistical significance was calculated using a one-way ANOVA with Tukey's multiple comparison. (**p*<0.05; ***p*<0.001; ****p*<0.0001).

Supp. Fig. 10

A

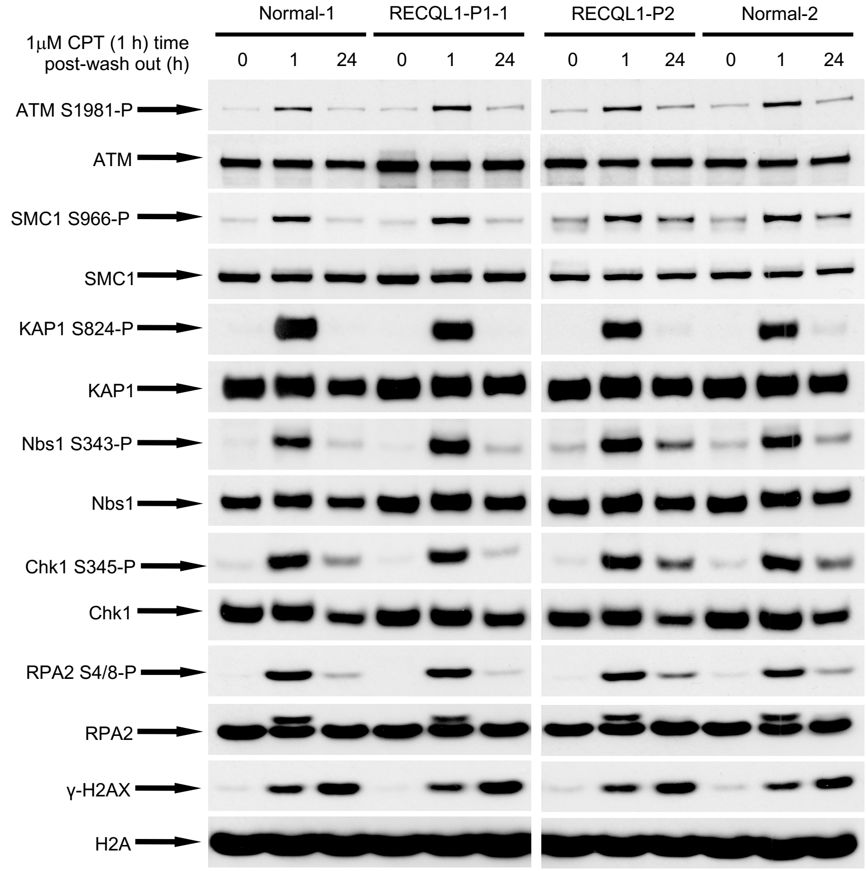

B

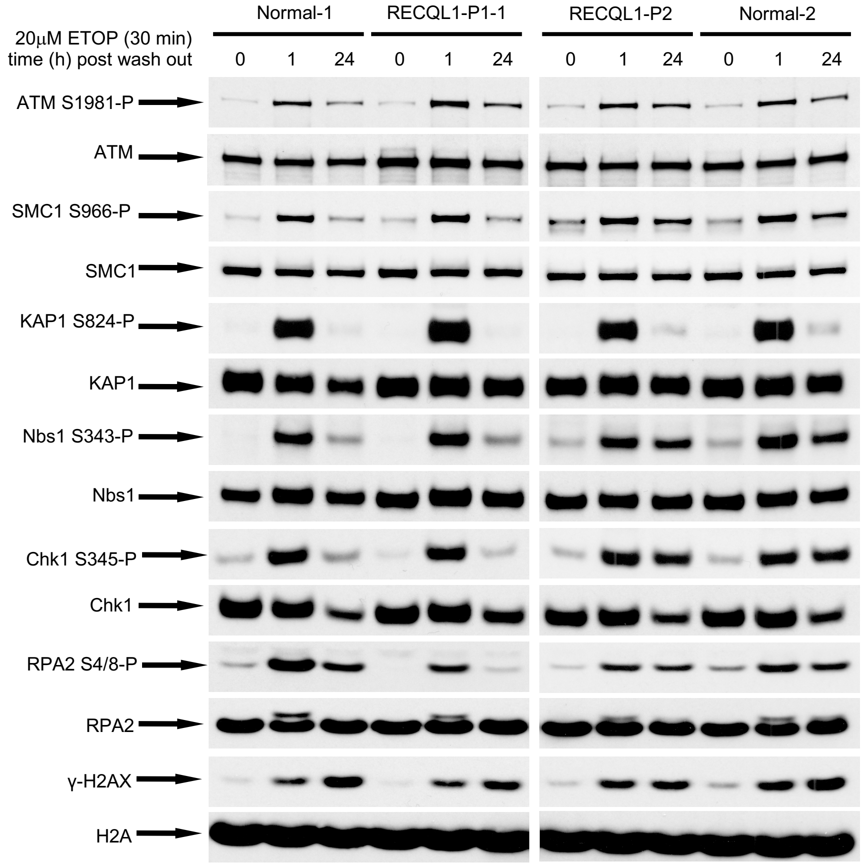

Supplementary Figure 10. *RECQL* mutant patient cell lines do not exhibit any defects in ATM-dependent DNA damage signalling following exposure to CPT and ETOP. (A) Western blot on cell extracts derived from LCLs from two normal individuals (Normal-1 and Normal-2) and two *RECQL* mutant patients (RECQL1-P1-1 and RECQL1-P2) before and after exposure to 1 μM CPT for 1 h. Following removal of CPT from cells, timepoints were taken1 h and 24 h post-CPT exposure. Antibodies used for Western blotting are indicated. Phospho-specific antibodies are denoted with the position of the phosphorylated residue followed by a 'P'. Antibodies to non-phosphorylated proteins were used to control for protein loading. (B) Western blots were carried out as in (A), with the exception that cells were exposed to 20 μM ETOP for 30 min and then removed. All Western blots using phospho-specific antibodies were generated from cutting the same nitrocellulose filter into strips and blotting with the antibodies indicated. All Western blots using antibodies to non-phosphorylated proteins were generated by running the same samples in parallel on a separate gel, cutting the nitrocellulose filter into strips and blotting with the antibodies indicated.

Supp. Fig. 11

A

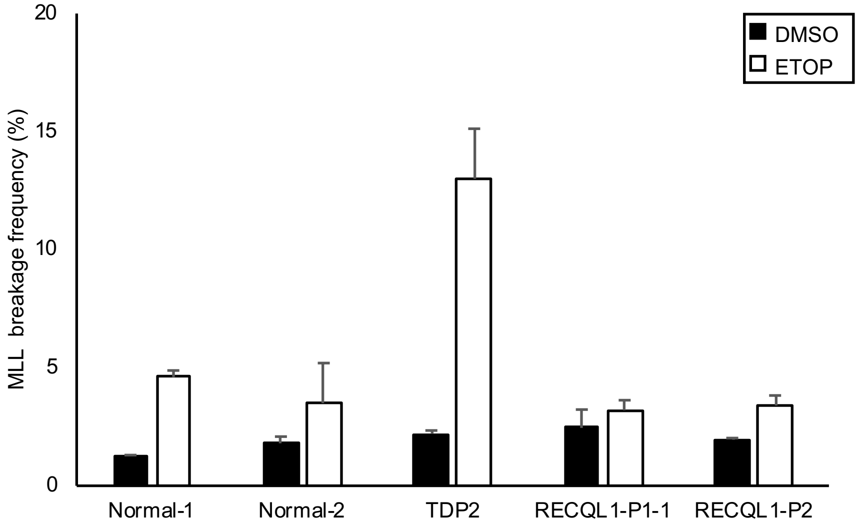

B

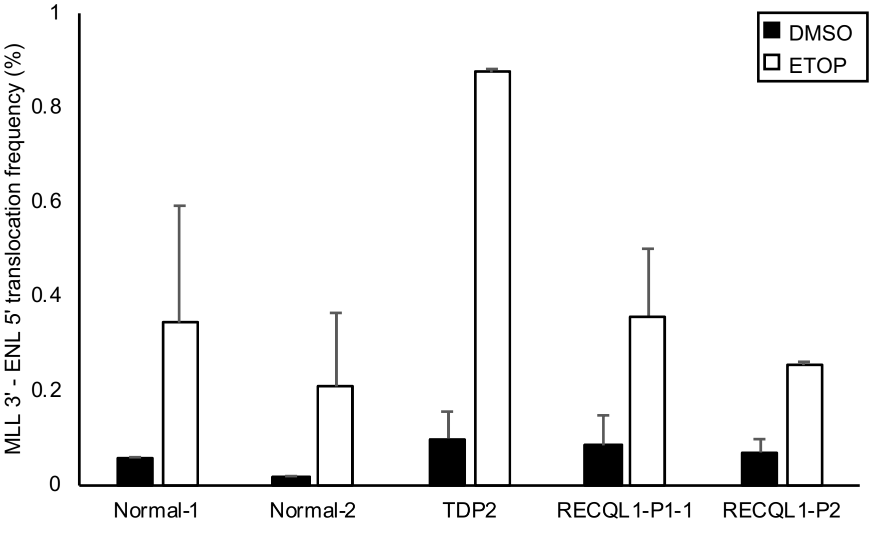

Supplementary Figure 11. *RECQL* mutant patient cell lines do not exhibit increased breakage and translocation of the *MLL* locus following exposure to etoposide. Quantification of chromosome breakage and *MLL-ENL* translocations in RECQL1 mutant LCLs using C-Fusion 3D. LCLs were treated with 20 µM of Etoposide for 4 h and released from damage stimulus for two days. Chromosome breakage **(A)** and translocation **(B)** frequencies were calculated by combining FISH and high-throughput imaging as in (25). A minimum of 4000 nuclei per condition was analyzed. Bar charts show the mean and SD of two independent experiments carried out in triplicate.

Supp. Fig. 12

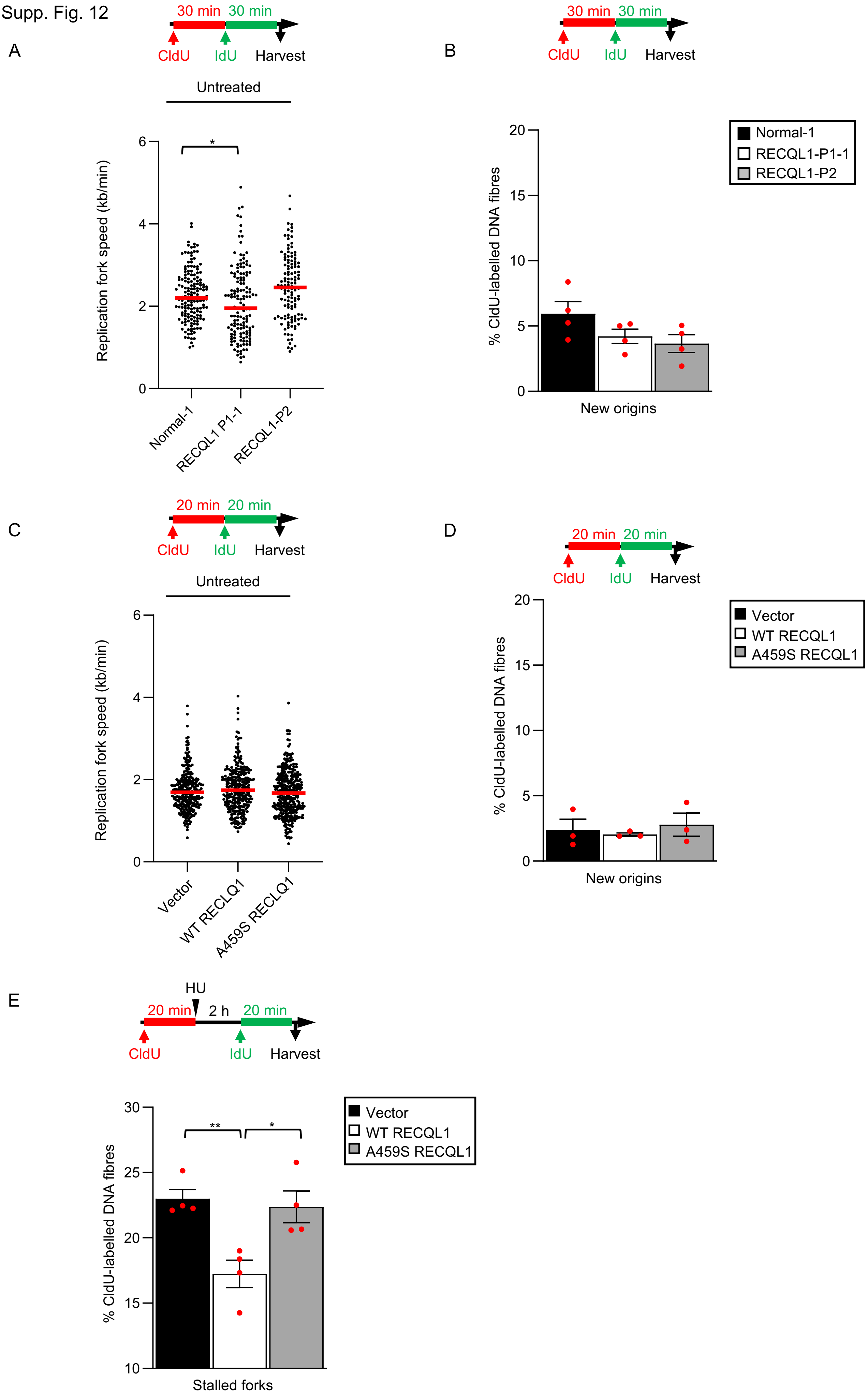
A
30 min
30 min
CldU
IdU
Harvest
Untreated
Replication fork speed (kb/min)
*
0
2
4
6
Normal-1
RECQL1 P1-1
RECQL1-P2
B
30 min
30 min
CldU
IdU
Harvest
% CldU-labelled DNA fibres
0
5
10
15
20
New origins
Normal-1
RECQL1-P1-1
RECQL1-P2
C
20 min
20 min
CldU
IdU
Harvest
Untreated
Replication fork speed (kb/min)
0
2
4
6
Vector
WT RECLQ1
A459S RECLQ1
D
20 min
20 min
CldU
IdU
Harvest
% CldU-labelled DNA fibres
0
5
10
15
20
New origins
Vector
WT RECQL1
A459S RECQL1
E
HU
20 min
2 h
20 min
CldU
IdU
Harvest
% CldU-labelled DNA fibres
10
15
20
25
30
**
*
Stalled forks
Vector
WT RECQL1
A459S RECQL1

Supplementary Figure 12. *RECQL* mutant patient cell lines do not exhibit spontaneous abnormalities with replication fork speed or new origin firing. (**A**) LCLs were sequentially labelled with CldU and IdU as shown. The length of the CldU and IdU tracks of dual labelled DNA fibres were measured and converted to kb and then the replication fork speed was calculated using the CldU/IdU incubation time. The median of three independent experiments is shown (red line). The replication fork speed was calculated for a minimum of 40 forks per cell line per experiment. Statistical significance was calculated using a Kruskal-Wallis test with Dunn's multiple comparison. (**B**) LCLs were sequentially labelled with CldU and IdU as shown. Newly fired replication forks (IdU only) were quantified. The mean of four independent experiments is shown. A minimum of 200 forks were counted per condition. Statistical significance was calculated using a one-way ANOVA with Tukey's multiple comparison. (**C**) Complemented RECQL1-P1-1 patient fibroblasts were sequentially labelled with CldU and IdU as shown. The median of three independent experiments is shown (red line). The replication fork speed was calculated for a minimum of 100 forks per cell line. Statistical significance was calculated using a Kruskal-Wallis test with Dunn's multiple comparison. (**D**) Newly fired replication forks (IdU only) were quantified in cells from (C). The mean of three independent experiments is shown. A minimum of 200 forks were counted per condition. Statistical significance was calculated using a one-way ANOVA with Tukey's multiple comparison. (E) HeLa RECQL1 CRISPR knockout cells complemented with empty vector, WT or mutant (p.A459S) RECQL1 were labelled with CldU, IdU and exposed to HU as shown. Stalled forks were quantified. The mean of four independent experiments is shown. A minimum of 250 forks were counted per condition. Statistical significance was calculated using a one-way ANOVA with Tukey's multiple comparison. (*$p<0.05$; **$p<0.001$; ***$p<0.0001$).

Supp. Fig. 13

A

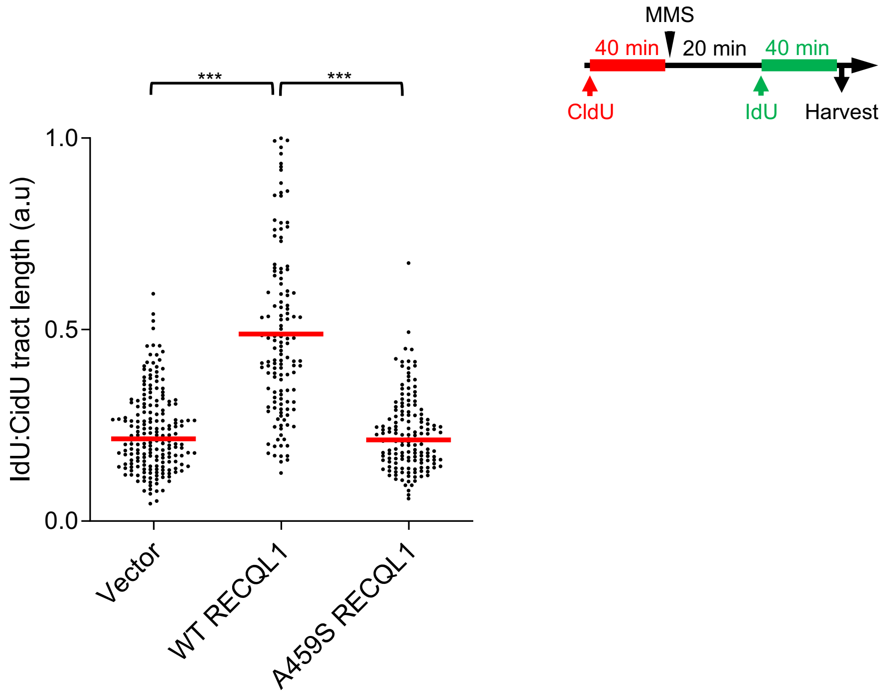

B

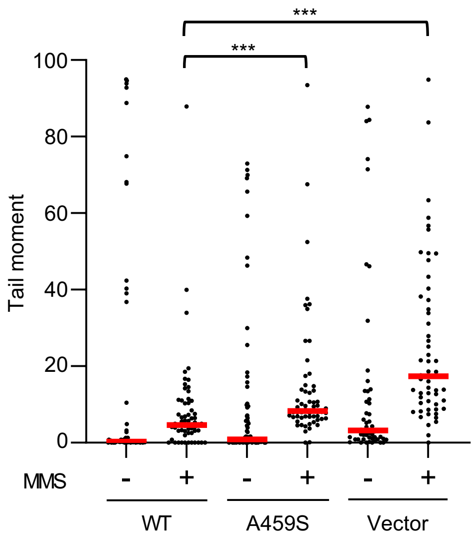

C

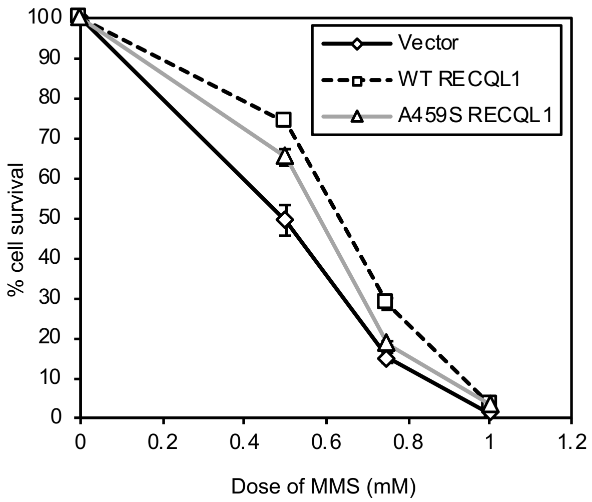

Supplementary Figure 13. DNA replication defects and increased genome instability in cells expressing the RECQL1 p.459S mutant protein following MMS exposure. (**A**) HeLa RECQL1 CRISPR knockout cells complemented with empty vector, WT or mutant (p.A459S) RECQL1 were labelled with CldU, exposed to 0.02% MMS as shown and then incubated in IdU. The IdU:CldU ratio was calculated for a minimum of 150 forks per condition from three independent repeats. The IdU:CldU ratio represents the efficiency of replication fork restart following removal of MMS. Statistical significance was calculated using a Kruskal-Wallis test with Dunn's multiple comparison. (**B**) HeLa RECQL1 CRISPR knockout cells complemented with empty vector, WT or mutant (p.A459S) RECQL1 were exposed to 0.01% MMS for 1 h. Cells were harvested and subjected to neutral comet assay. A dot plot showing the tail moment of individual samples is shown. The mean of three independent experiments is shown. 50-70 comets were analysed for each sample per experiment using the OpenComet plugin in ImageJ. Statistical significance was calculated using a Kruskal-Wallis test with Dunn's multiple comparison. (**C**) Colony survival assay using complemented HeLa RECQL1 CRISPR knockout cells exposed to increasing doses of MMS. The survival of untreated cells was set as 100% and the survival following MMS exposure is shown as percent survival relative to the untreated cells. The data represents the mean of three independent experiments AUC: Vector (mean±SEM: 47.39±1.907); WT (mean±SEM: 60.32±0.7589); A459S (mean±SEM: 54.49±1.036). Vector vs WT vs A459S AUC one-way ANOVA: p<0.0014. (*p<0.05; **p<0.001; ***p<0.0001).

Supplementary Materials and Methods

DNA binding assays. DNA binding by RECQL1 proteins was determined by electrophoretic mobility shift assay (EMSA) as previously described (19). Briefly, for conventional assays RECQL1-WT and RECQL1-A459S proteins were diluted in dialysis buffer, and specified concentrations were incubated with the 19-bp forked duplex DNA substrate (0.5 nM). DNA binding incubations were carried out in 20 mM Tris (pH 7.4), 10 mM KCl, 5 mM $MgCl_2$, 10% glycerol, 80 ng/µl BSA with or without 5 mM ATPγS, as specified. After incubating binding mixtures for 15 min at 37 °C, 4 µl of loading dye (26.7% glycerol, 0.02% bromphenol blue and 0.02% xylene cyanol) was added and the binding mixtures were electrophoresed on native 5% polyacrylamide gels at 200 V for 2 h. The apparent DNA binding dissociation constants (K_d) were calculated as described previously (19).

For competitive inhibition DNA binding assays, DNA binding mixtures containing 10 nM RECQL1-WT or RECQL1-A459S proteins were pre-incubated with 0.5 nM radiolabeled 19-bp forked DNA substrate for 8 min at 37 °C, followed by the addition of increasing concentrations (1.25 - 50 nM) of unlabeled oligonucleotide and incubated for an additional 7 min. The DNA binding mixtures were resolved by gel electrophoresis and analyzed as described above.

ATPase assays to determine kinetic constants k_{cat} and K_m for RECQL1 proteins. RECQL1 ATPase assays were performed as previously described (18). Briefly, for experiments to determine k_{cat}, ATPase reactions (30 µl) contained 20 mM Tris–HCl (pH 7.5), 5 mM $MgCl_2$, 10 mM KCl, 10% glycerol, 80 µg/ml BSA, M13mp18 ssDNA effector (32 µM nucleotide), a trace amount of [γ-^{32}P]ATP (~2 nM) mixed with 1 mM cold ATP, and 20 nM RECQL1 (WT or A459S). The reactions were initiated by adding RECQL1 protein and incubated at 37 °C. Samples (5 µl) were removed at 5-10 min intervals and quenched by the addition of STOP solution (6.7 mM ATP, 6.7 mM ADP, 33.3 mM EDTA) and spotted on PEI-Cellulose thin layer chromatography sheets (10 x 20 cm). The solvent (1 M formic acid, 0.8 mM LiCl) was allowed to migrate 0.5 cm from the top of the sheet, and sheet was exposed to a phosphoimager screen for 1 h and scanned. ImageQuant TL software was used to quantitate hydrolyzed ATP. Less than 10% of the ATP substrate was consumed in the reaction over the entire time course of the experiment. The k_{cat} values were expressed as the mean of at least four independent determinations with standard deviation.

ATPase assays to determine K_m were similar to those for assays to determine k_{cat} with a few exceptions. ATPase assay reactions (20 µL) contained the same buffer components and M13mp18 ssDNA effector (32 µM nucleotide), but increasing concentrations of ATP (31.3 µM - 2 mM [γ-^{32}P]ATP:ATP mixture in 1:500,000 ratio), and 40 nM RECQL1 (WT or A459S). The reactions were initiated by adding RECQL1 and incubated at 37 °C. Samples (5 µl) were removed at 0 and 15 min and quenched by the addition of STOP solution and processed as described above. The K_m values were expressed as the mean of at least three independent determinations with standard deviation.

Size exclusion chromatography. Purified recombinant RECQL1-WT or RECQL1-A459S (3 µM, 0.5 ml) was applied to a 24 ml Superdex-200 size exclusion column (GE Healthcare) using an AKTA FPLC (GE Healthcare) that was equilibrated in Buffer A (25 mM Tris-HCl (pH 7.5), 10% glycerol, 0.15 M NaCl, 1 mM EDTA, 0.5 mM DTT) or Buffer B (20 mM Tris-HCl (pH 7.5), 150 mM KCl, and 1 mM DTT) in the presence or absence of 1 mM ATP. The purified RECQL1 was then eluted from the column in two column volumes of either Buffer A or B. The column was run at a rate of 0.1 ml/min, and 0.5-ml fractions were collected. Proteins were detected using a UV detector.

FACS analysis. Cells were fixed in 70% ethanol and stored at -20 °C. Cells were then washed twice in PBS prior to addition of 20 µg/ml RNase (Sigma Aldrich) and 10 µg/ml propidium iodide (PI) (Sigma Aldrich). Relative DNA content was determined using a CytoFLEX flow cytometer (Beckman Coulter).

Colony survival assay. Cells were plated (1000 cells/well) on 6-well plate and allowed to adhere for 4 h before MMS treatment. After 1 h of MMS treatment, cells were washed 3 times with 1X PBS and allowed to form colonies in drug-free RPMI-1640 medium for 10 days. For CPT/ETOP sensitivity, cells were continuously exposed to the indicated doses of CPT/ETOP and allowed to form colonies for 12 days. Colonies were fixed with 100% methanol, stained with 0.5% crystal violet and quantified.

Neutral comet assay. DNA breaks were determined by comet assay (single-cell gel electrophoresis) under non-denaturing conditions according to the Comet SCGE assay kit (Enzo, ADI-900-166) protocol. Briefly, cells grown in 6-well plate were treated with 0.01% MMS for 1 h to induce DNA breakage. Cell were trypsinized, counted and resuspended at 1 x 10^5 cells/ml in ice cold 1X PBS (Ca^{2+} and Mg^{2+} free).

Cells were combined with molten low-melting agarose (at 37 °C) at a ratio of 1:10 (v/v) and 75 µl of cells/agarose suspension was evenly spread onto CometSlide (Enzo). After solidifying the agarose, slides were immersed in 1X lysis buffer (Enzo) at 4 °C for 1 h, and subsequently subjected to horizontal gel electrophoresis using 1X ice cold TBE buffer with 21 volts for 20 minutes at 4 °C. Slides were immersed in 70% ethanol for 5 minutes and left for air drying. Nuclei were stained with 10X CYGREEN dye (Enzo) and mounted using permount mounting media (Fisher Scientific). Comet tails were visualized using GFP filter of Zeiss Axio Observer.Z1 fluorescence microscope with 10X objective lens. Tail moment was analyzed using OpenComet analysis tool (S1) in ImageJ.

Preparation of reversed and replication fork structures. To prepare the reversed fork and replication fork substrates, 10 pmol of oligonucleotide B (5'-CGGGTGTCGGGGCGCATGACACTATGCGGCATCAGAGCAGATTGTACTGAAGGTCAACATGTTGTAAATATGCAGCTAAAG-3') was labelled with [γ^{32}P]ATP using T4 polynucleotide kinase (T4PNK) for 40 min at 37 °C followed by 20 min of heat inactivation at 65°C. The oligo was passed through a prespun microspin G25 column (Cytiva) for 2 min at 700 *x g* to remove unincorporated [γ^{32}P]ATP. The radiolabeled oligonucleotide B was annealed to 10 pmol of oligonucleotide A (to make the reversed fork, 5'-CTTTAGCTGCATATTTACAACATGTTGACCTTCAGTA/isodC/AATCTGCTCTGATGCCGCATAGTGTCATGCCAGAGCTTTGTAC-3') or oligonucleotide D (to make the replication fork, 5'-TCAGTACAATCTGCTCTGATGCCGCATAGTATCATGCGCCCCGACACCCG-3') in 50 µl annealing buffer (10 mM Tris, pH 7.5 and 50 mM NaCl) by heating to 95°C for 5 min followed by slow cooling overnight. In other overnight annealing reactions, oligonucleotides C (5'-GTACAAAGCTCTGGCATGATACTATGCGGCATCAGAGCAGATT-3') and D (50 pmol each) were annealed using the same annealing conditions to prepare the other half of the reversed fork, while oligonucleotides A and C (also 50 pmol each) were annealed to prepare the rest of the replication fork. Just prior to running the fork restoration assay, 1 pmol of radiolabelled AB fork was annealed to 2 pmol of CD fork and in the presence of 10 mM Tris pH 7.5, 50 mM NaCl and 5 mM $MgCl_2$ at 37 °C for 30 min followed by 30 min at RT to generate the reversed fork structure. To prepare the replication fork, the radiolabelled BD fork was annealed to the AC fork under identical conditions to the reversed fork

preparation. Both the reversed and replication fork substrates were used immediately after annealing in the fork restoration assay.

C-Fusion 3D high-throughput imaging. LCLs were challenged with 20 µM Etoposide or DMSO for 4 h and released into fresh medium for 48 h prior to fixation. Fixing and permeabilization of the LCLs, incubation with the FISH probes for the *MLL (KMT2A)* and *ENL (MLLT1)* genes and imaging were carried out as previously described (25).

Metaphase spreads and sister chromatid exchange labelling. For SCEs, LCLs were incubated with 10 µM BrdU for 48 h before incubating with 0.2 µg/ml demecolcine for 3 h. Cells were then resuspended in 0.075M KCl, incubated at 37 °C for 30 min and fixed in ethanol/acetic acid (3:1). Metaphases were dropped onto microscope slides, incubated in 10 µg/ml Hoescht for 20 min and exposed to UVA light for 1 h in 20× SSC buffer. Slides were incubated in 20× SSC buffer for 1 h at 60 °C and stained with 5% Giemsa.

Generation of RECQL1 CRISPR knockout HeLa cells. RECQL null HeLa cells were generated using the commercially available RECQL1 CRISPR/Cas9 KO plasmid (sc-402845, Santa Cruz Biotechnology). HeLa cells were transfected with the CRISPR/Cas9 KO plasmid using Lipofectamine LTX (Invitrogen) according to the manufacturers protocol and then sorted by FACS-sorted into 96-well plates using a BD FACSAria II instrument. Resulting colonies were screened by Western blotting.

Statistics. All analyses were done with Graphpad Prism version 9 (GraphPad Software, San Diego, CA, USA). Parametric and nonparametric data are presented as mean (standard error of the mean) and median (interquartile range), respectively. One-way and two-way analysis of variance with Tukey correction, Kruskal-Wallis test with Dunn intergroup comparison, or unpaired *t* test and Mann-Whitney test were used to compare across groups for parametric and nonparametric data, respectively, as appropriate. A *p* value of less than 0.05 was taken as the threshold of statistical significance; $^{*}p<0.05$, $^{**}p<0.01$, $^{***}p<0.001$.

Supplemental Reference

1. Gyori BM, et al. OpenComet: an automated tool for comet assay image analysis. *Redox Biol*. 2014;2:457–465.
